# Supplementary material for: How effective and cost-effective are innovative combinatorial technologies and practices for supporting older people with long-term conditions to remain well in the community? An evaluation protocol for an NHS Test Bed in North West England
Source: BMJ Open. 2018 Feb 28;8(2):e017268. doi: 10.1136/bmjopen-2017-017268 (PMC5855480; doi:10.1136/bmjopen-2017-017268)
Supplement: Supplementary file 1 [file bmjopen-2017-017268supp001.pdf]

Appendix 1

Participant payment

Participants taking part in the qualitative elements of the study will receive a voucher of £20 as a thank you in recognition of the time they have given to taking part in the evaluation. In the quantitative elements of the evaluation we are unable to offer any incentive because of the large numbers of participants.

Data storage

Personal data is being stored on a password safe university computer accessible only to the researchers. The research data is stored in an encrypted file using the software TrueCrypt.

All data is being stored securely in line with the Data Protection Act [15] and will be kept no longer than necessary.[16] Written data will be kept for a maximum of 10 years in a secure cabinet. Any electronic copies of data will be kept on the University server, which is a password protected system and will be encrypted. If electronic data requires transfer, this is done on an encrypted USB device. Any emails sent from participants (e.g. electronic diaries) are sent to a password secured email address that has been set up for the purpose of the study. Emails are saved electronically with any other electronic copies of data, with the email then being deleted. Where emails and interview/focus group data are printed for the purposes of analysis, they are stored with other written data in a secure cabinet. At the end of the study, the email account will be deleted. The paper copies of any participant contact details will be scanned and stored electronically, and paper copies destroyed.

Participant benefit

Participants have the direct benefit of using the technology during the two years of the Test Bed; however the Test Bed team recognise the need to make clear for those in Cohorts 1 and 4 that this is time-limited. Patients (particularly those in cohorts 2 and 3) for whom the intervention is a text messaging system or an app, will have access to these beyond the six month timeframe of the Test Bed programme. Given a key objective of the service is to enhance patient activation and ability to self-care, it is also anticipated that potential ongoing benefits will support older people with chronic health problems to remain in their own homes longer.

Patients and health professionals also have an opportunity to share directly with researchers their experiences of using and implementing this model of care. In this way, all participants can take an active part in shaping current and future healthcare services in place for older people with chronic conditions or dementia who are living at home.

Potential risk to participants

For patients participating in Phase 1 of the evaluation, there are no anticipated risks from participation in the project. All validated tools are validated for completion by proxy, and patients and family

members are therefore made aware that the patient can be supported to complete the surveys if required.

For patients taking part in Phase 2 of the evaluation research, no specific risks are anticipated. However, the following considerations have been made:

1. It is possible that a patient may become distressed by the presence of the researcher in their home during the observational interviews. To avoid this risk, the researcher will take care to check with the patient at the beginning of the interview to ensure they are happy to continue taking part in the study. The researcher will also do this at the end of the session and for the second interview.

2. If a patient should become distressed for any reason during an observational interview, the researcher will provide the patient with the contact details of different support networks for older people, as outlined in the participant information sheet. A member of the Test Bed hub will also be alerted should this be necessary, in order to assess whether a visit from a member of the hub is required.

Participants involved in the action learning meetings and the focus groups will be reminded that discussion in these settings is confidential and information shared (other than the shared and agreed action learning) should not be discussed with others outside of the meeting.

Withdrawal

Participants may choose to leave the study at any time and are assured that their data can be withdrawn up to four weeks after collection. The rationale for this four week time period is that, beyond four weeks, analysis will have begun. Those participating in focus groups are advised that, although every effort will be undertaken to remove their data should they request it during the timeframe, this may not always be possible. This is due to difficulties in identifying/attributing specific comments to individuals and because focus group data is often the outcome of group discussion, making it hard to distil to whom individual thoughts/comments should be attributed.

Researcher risk

There is limited risk to the quantitative researchers as they have very limited direct engagement with the participants (survey completion is largely through technology or telephone).

The qualitative researcher is interviewing participants in their own homes and is experienced in undertaking this type of fieldwork. The researcher is adhering to the University's lone worker policy. In order to minimise any potential risks, for each home visit conducted, the researcher identifies a 'buddy' (a member of staff within the University) with whom to remain in contact with: before the interview commences; when the interview has been terminated; and when the researcher has left the patient's home. The buddy is informed of the time and place of the interview, along with a contact telephone number.

Researchers within the team have the opportunity to debrief with other members of the team if required and have full access to counselling services if necessary.

References

15 Data Protection Act (1998) Data Protection Act. Available at: http://www.legislation.gov.uk/UKPGA/1998/29/contents [Accessed on 17 February 2017].

16 Bryman A. Social Research Methods, Sixth Edition. Oxford, UK: Oxford University Press 2016.
