# Supplementary material for: How effective and cost-effective are innovative combinatorial technologies and practices for supporting older people with long-term conditions to remain well in the community? An evaluation protocol for an NHS Test Bed in North West England
Source: BMJ Open. 2018 Feb 28;8(2):e017268. doi: 10.1136/bmjopen-2017-017268 (PMC5855480; doi:10.1136/bmjopen-2017-017268)
Supplement: Author's manuscript [file bmjopen-2017-017268.draft_revisions.pdf]

BMJ Open is committed to open peer review. As part of this commitment we make the peer review history of every article we publish publicly available.

When an article is published we post the peer reviewers' comments and the authors' responses online. We also post the versions of the paper that were used during peer review. These are the versions that the peer review comments apply to.

The versions of the paper that follow are the versions that were submitted during the peer review process. They are not the versions of record or the final published versions. They should not be cited or distributed as the published version of this manuscript.

BMJ Open is an open access journal and the full, final, typeset and author-corrected version of record of the manuscript is available on our site with no access controls, subscription charges or pay-per-view fees (http://bmjopen.bmj.com).

If you have any questions on BMJ Open's open peer review process please email editorial.bmjopen@bmj.com

BMJ Open

New Models of Care – Lancashire and Cumbria Innovation Alliance Test Bed Evaluation of Fylde Coast Vanguard/Better Care Together with Technology: study protocol

Journal:	*BMJ Open*
Manuscript ID	bmjopen-2017-017268
Article Type:	Protocol
Date Submitted by the Author:	11-Apr-2017
Complete List of Authors:	Varey, Sandra; Lancaster University, Division of Health Research<br>Hernândez, Alejandra; Lancaster University<br>Palmer, Tom; Lancaster University, Department of Mathematics and Statistics ; University of Warwick, Warwick Medical School, Division of Health Sciences<br>Mateus, Ceu; University of Lancaster, Division of Health Research<br>Wilkinson, Joann; Lancaster University<br>Dixon, Mandy; Lancaster University<br>Milligan, Christine; Lancaster University
Keywords:	Health technologies, older people, long-term conditions, patient activation, self-care

SCHOLARONE™
Manuscripts

Title: New Models of Care – Lancashire and Cumbria Innovation Alliance Test Bed Evaluation of Fylde Coast Vanguard/Better Care Together with Technology: study protocol

Sandra Varey[1], Alejandra Hernández[1], Tom Palmer[2], Céu Mateus[3], Joann Wilkinson[1], Mandy Dixon[4] and Christine Milligan[1]

1. Centre for Ageing Research, Division of Health Research, Lancaster University, Lancaster LA1 4YG, United Kingdom
2. Department of Mathematics and Statistics, Lancaster University, Lancaster LA1 4YG, United Kingdom
3. Health Economics, Division of Health Research, Lancaster University, Lancaster LA1 4YG, United Kingdom
4. Lancaster Health Hub, Lancaster University, Lancaster LA1 4YG, United Kingdom

Correspondence to:

Professor Christine Milligan

Centre for Ageing Research

Division of Health Research

Lancaster University

Lancaster LA1 4YG

United Kingdom

Email: c.milligan@lancaster.ac.uk

Telephone: +44 1524 592128

Word count: 4261

For peer review only - http://bmjopen.bmj.com/site/about/guidelines.xhtml

Abstract

Introduction:

The Lancashire and Cumbria Innovation Alliance (LCIA) Test Bed is a partnership between the UK National Health Service (NHS), industry (led by Philips) and Lancaster University. Through the implementation of a combination of innovative health technologies and practices, it aims to determine the most effective and cost effective ways of supporting frail older people with long-term conditions to remain well in the community. Among the Test Bed's objectives are to: improve patient activation and the ability of older people to self-care at home; reduce healthcare system utilisation; and deliver increased workforce productivity.

Methods and Analysis:

Patients aged 55 years and over are recruited to four cohorts defined by their risk of hospital admission, with long-term conditions including COPD, dementia, diabetes and heart failure. Interventions are determined on an individual basis, with a range of technologies available. The evaluation is adopting a two-phase approach: Phase 1 includes a bespoke patient survey and a mass matched control analysis; and Phase 2 is using observational interviews with patients, and weekly diaries, action learning meetings and focus groups with members of staff and other key stakeholders. Phase 1 data analysis consists of a statistical evaluation of the effectiveness of the intervention and a health economic analysis of its costs and associated cost changes. Phase 2 data will be analysed thematically with the aid of Atlas.ti qualitative software. The evaluation is located within a logic model framework, to consider the processes, management and participation that may have implications for the Test Bed's success.

Ethics and Dissemination:

The LCIA Test Bed evaluation has received ethical approval from the Health Research Authority and Lancaster University's Faculty of Health and Medicine Research Ethics Committee. A range of dissemination methods are adopted including deliberative panels with key stakeholders to validate findings and develop outcomes for policy and practice.

Word count: 300

Strengths and limitations of this study

- The evaluation is considering both the effectiveness, in terms of measures such as hospital admissions, and cost effectiveness of the Test Bed
- The protocol evaluates the potential for patient activation through health technology
- Observational interviews with patients in their own homes enable insights into how older people engage with the technology
- For both the Test Bed participants and controls the evaluation has to use proxy measures of the costs incurred to the NHS
- Due to the timescale of the overall programme, it is not possible to assess the longer-term impact post-intervention

For peer review only - http://bmjopen.bmj.com/site/about/guidelines.xhtml

Key words

Health technologies; older people; long-term conditions; patient activation; self-care.

INTRODUCTION

In the UK, increasing numbers of older people are living with long-term conditions including diabetes, chronic obstructive pulmonary disease (COPD), heart failure and dementia.[1] This is contributing to increased pressure on health and social care systems including accident and emergency services, which in turn increases demand on the number of hospital beds.[2] At the same time, local authorities are experiencing a reduction in the funding available to provide social care.[3]

Concerns about this so-called care-gap and our future ability to cope with the growing numbers of older people with long-term conditions underpins governmental drivers to develop new models of care that are cost-effective and which facilitate older people's ability to better self-manage their care needs at home. The NHS England Test Bed programme has thus been designed to encourage the trialling of new models of care that are supported by health technologies across a number of areas in the UK.

This paper reports on the protocol for the evaluation of one such programme, the Lancashire and Cumbria Innovation Alliance (LCIA) Test Bed in the north-west of England. The programme commenced on 1st April 2016 and is due to be completed on 31st March 2018.

The LCIA Test Bed initiative

The LCIA Test Bed[1] is a partnership between the NHS, industry (led by Philips) and Lancaster University, and is one of seven Test Beds located across England. Two neighbouring Vanguard sites[2], part of the Lancaster Health Hub (an established NHS/University partnership comprising ten local organisations), will deliver the Test Bed. The Test Bed's aims and objectives are presented below.

Test Bed Aims:
To determine the most effective and cost effective ways of supporting frail older people with dementia and other long-term conditions (LTCs) to remain well in the community and avoid unnecessary hospital admissions.

Test Bed Objectives:
To use a combinatorial range of technologies and interventions to:

- better support frail older people, older people living with long-term conditions;
- improve patient activation and the ability of older people with a range of LTCs to self-care at home;
- improve health awareness and outcomes for older people with LTCs;
- reduce healthcare system utilisation and deliver increased workforce productivity.

---

[1] For more information, visit: https://www.england.nhs.uk/ourwork/innovation/test-beds/innovation-alliance/

[2] In 2015, the National Health Service in England selected 50 vanguard sites to take a lead on the development of new integrated care models designed to act as blueprints for the future of the NHS. Complete redesign of whole health and care systems are being considered. The hope is that the vanguards will provide inspiration to the rest of the health and care system across the UK (NHS England, 2017).[6]

For peer review only - http://bmjopen.bmj.com/site/about/guidelines.xhtml

Over two years, the Test Bed is implementing a combination of innovative technologies and practices aimed at supporting these aims and objectives. The clinical challenge is to reduce hospital admissions, create capacity, reduce overall costs, minimize other health and social care usage, and maximize patient outcomes for a targeted population of older people (defined in this Test Bed as those aged 55 years and over) who suffer a range of long term conditions including dementia. These LTCs present a major challenge for the area given its dispersed population, and innovative solutions are urgently required.

The main technology partner is Philips who, together with a number of small and medium sized enterprises (SMEs), social enterprises, and voluntary organisations, is working with the Test Bed to introduce a health technology-enabled supported self-care programme[3].

The new model of care includes services in the two Vanguard sites in the Fylde Coast and North Lancashire. The Fylde Coast Vanguard[4] is using a range of health services; one of these is Extensive Care, which brings together a range of services in the same location to achieve a co-ordinated team of healthcare professionals, with patients each allocated a wellbeing support worker.[4] In the North Lancashire Vanguard, Better Care Together sees healthcare professionals working together in partnership to help people manage their own health conditions.[5] As part of the LCIA Test Bed programme, these existing services are being combined with innovative technologies and support for older people with LTCs including COPD, Heart Failure, Diabetes and Dementia. For those with lower levels of risk in cohort 3 (see Table 1), asthma and hypertension will also be included. The model of care in this Test Bed is designed to empower patients to actively manage their health conditions and change behaviour through the provision of appropriate coaching and education.

A core part of the programme also focuses on increasing involvement of the third sector and community organisations in providing support for this new model of care with technology; and understanding the impact of the intervention on staff and key stakeholders.

Core outcomes for the evaluation

The Test Bed focuses on a combinational approach to technologies. Hence, rather than focusing on the impact of any single technology, the Test Bed evaluation is focusing on how health technologies as an addition to existing Vanguard services can improve and promote self-care at home and reduce hospital admissions for older people with LTCs.

The evaluation is focusing on three primary outcomes:

1. The extent to which supported self-care telehealth technology can improve patient outcomes, through reduced hospital admissions, medication, GP visits, home visits and community service use;

2. The extent to which supported self-care telehealth technology can improve patient activation for frail older people with LTCs (to include increased sense of empowerment, improved quality of life, service satisfaction, enhanced knowledge, skills and confidence to self-manage care);

[3] Cambridge Cognition Limited; Good Things Foundation; Intelesant Limited; MKS Solutions Limited; Simple Shared Healthcare Limited; and uMotif Limited.
[4] The Fylde Coast Vanguard encompasses NHS Blackpool CCG, NHS Fylde & Wyre CCG and Blackpool Teaching Hospitals.

For peer review only - http://bmjopen.bmj.com/site/about/guidelines.xhtml

3. The cost effectiveness of the intervention and how it might be scaled up to provide better value for patients and taxpayers.

Secondary outcomes include:

1. Identification of the strengths and weaknesses of the intervention and how these may be built on or addressed;

2. Assessment of patient/staff perceptions of how the intervention improves on existing services and makes best use of voluntary and community services;

3. Assessment of the impact of the intervention on the workforce in terms of communication between care teams, productivity, capacity, co-ordination of care, and work satisfaction.

To achieve this, we are:

a) Using quantitative and qualitative measures to examine benefits to patients and patient outcomes as identified above;

b) Undertaking an economic evaluation of the impact of adding a combination of technologies to support and promote self-care in the provision of healthcare in comparison to standard care;

c) Drawing on a Logic Model to demonstrate how to construct the service as a scalable model that might provide better value to patients and taxpayers.

METHODS AND ANALYSIS

Ethical approvals

The evaluation is adopting a two-phase approach: Phase 1 focuses on service evaluation and is quantitative in its design; while Phase 2 focuses on more in-depth understandings of patient and staff experiences of the service through qualitative research.

These two distinct stages have received ethical approval through two different routes: Phase 1 was deemed by the Health Research Authority (HRA) to be service evaluation not requiring NHS ethics approval. However, it was submitted and has been awarded ethical approval from Lancaster University's Faculty of Health and Medicine Research Ethics Committee (Reference: FHMREC16025). Phase 2 was deemed by HRA to be research and thus was submitted and has received ethical approval from the HRA (IRAS Project ID: 208395). Further details about the two phases are provided below.

Phase 2 of the evaluation has been adopted onto the National Institute for Health Research portfolio.[5]

Sampling and recruitment

[5] NIHR Portfolio reference number is the same as the IRAS number (IRAS Project ID 208395)

For peer review only - http://bmjopen.bmj.com/site/about/guidelines.xhtml

Patients are being recruited to four cohorts defined by their risk of hospital admission. The long-term conditions are noted above and are outlined in Table 1.

Table 1: Recruitment criteria for the four LCIA Test Bed cohorts

Cohort	Age	Risk of hospital admission	Long-term conditions
Cohort 1	Aged 55 years or over	>25%	COPD, HF
Cohort 2		>10% and <25%	
Cohort 3		<10%	Diabetes, Asthma, CHD, Hypertension
Cohort 4		N/A	Early stage dementia (ACE-III assessment tool)

For peer review only

Participants for cohorts 1, 2 and 3 are being recruited to the Test Bed through the Vanguard services to which they are already assigned. As illustrated in Table 1, Cohort 4 is focusing specifically on patients with early stage dementia, as identified through the ACE-III assessment tool. Potential participants for cohort 4 are being recruited through the Memory Assessment Service (MAS) in the Fylde Coast and North Lancashire. Those who have received a recent diagnosis of mild dementia by a consultant psychiatrist following their comprehensive assessment with the MAS are deemed as having fulfilled the entry criteria of mild dementia. For these patients, the ACE-III or Mini-ACE has been completed as part of their cognitive assessment at the MAS clinic, the results of which have aided the formulation of the dementia diagnosis.

All patients consenting to the take part in the Test Bed and to receiving the intervention are required to consent to participate in Phase 1 of the service evaluation. Participation in the qualitative Phase 2 of the evaluation is optional. Patients indicating an interest in participating in Phase 2 are being consented separately through a two-phase consent process.

The sample size for recruitment to each cohort has been informed by population and risk data for the area, along with the methodology used in a previous study.[7,8] As Table 2 illustrates, the overall aim of the Test Bed is to recruit 1600 patients; however, given the anticipated challenges of recruitment and potential attrition due to the nature of the patient group, we estimate an overall recruitment and retention figure of 60% (overall N=960).

Table 2: LCIA Test Bed cohort sample sizes (*combined over a population of app. 310,000 people)[6]

[6] See Acknowledgements

For peer review only - http://bmjopen.bmj.com/site/about/guidelines.xhtml

Cohort	Percent of the total population*	Total patients	Percent of frail and elderly patients	Percent of patients with dementia	Number of patients estimated to be appropriate for the service	Percent of appropriate patients within cohort that will be recruited within the Test Bed period	Minimum number of patients recruited within Test Bed period	Estimated number of patients by the end of the Test Bed period
**1** (age>55, risk>25% & 1 LTC)	1.70%	5,262	20%	3.5%	1,737	30%	521	500
**2** (age>55, 10%>risk<25 % & 1 LTC)	3.00%	9,286	4%	1.1%	3,064	15%	460	500
**3** (age>55, 10%<risk 1 of a broader set of LTCs)	9.20%	28,477	2%	1.6%	9,398	5%	470	500
**4** (diagnosis of dementia)	0.79%	2,430		100.0%	810	10%	81	100

Allowing for a phased increase in recruitment over the intervention period, the overall recruitment is estimated at approximately 60-70% of the figures identified in Table 2. This will provide sufficient data from cohorts 1 and 2 to demonstrate a change in hospital admissions (a primary outcome). It is unlikely that the evaluation will be able to demonstrate statistical significance for cohorts 3 and 4 for this outcome, although other measures of wellbeing, patient activation, and effectiveness of the technologies will be assessed. The secondary outcomes are being supported by observational interviews across all four cohorts.

The intervention

The patient interface varies across the four cohorts, and the technologies available to people within the different cohorts are presented in Table 3. A patient's intervention is determined on an individual basis and is informed by a number of factors, including health condition(s), personal preferences and other needs. Some patients are using different technologies at different periods of time within the six month intervention.

Table 3: Technologies available to each cohort

Cohort	Company	Technology	Description
1 & 4	Philips	Motiva[7]	A telehealth platform which operates through a tablet or TV set top box interface that connects wirelessly to a range of tele-monitoring equipment in the home (e.g. wireless weighing scales, blood pressure meters, pulse oximeters and thermometers).
	MKS Solutions Limited	Speakset[8]	A video calling system that connects a health professional with a patient through the patient's TV in their own home.

[7] http://www.philips.co.uk/healthcare/solutions/enterprise-telehealth/home-telehealth
[8] http://www.speakset.com

For peer review only - http://bmjopen.bmj.com/site/about/guidelines.xhtml

	NHS Stoke-on-Trent Clinical Commissioning Group's Licence	Florence/NHS Simple[9]	A short message communication software providing a reminder/communication text messaging service for patients.
1, 2 & 3	Cambridge Cognition Limited	CANTAB Mobile[10]	An assessment tool designed for healthcare professionals to identify the earliest signs of clinically significant memory impairment. The assessment comprises three tests: the Paired Associates Learning (PAL) test to assess episodic memory; the Geriatric Depression Scale (GDS) to identify signs of depression and an Activities of Daily Living questionnaire (ADL) to assess functionality in daily life.
2	Intelesant Limited	Intelesant[11]	A mobile app to support daily management of COPD through SMS text messaging alerts for coaching, prompts, and reminders. It incorporates the individual's action plan in response to changes in symptoms.
	Philips	Personal Blood Pressure Cuff[12]	A blood pressure monitor that works in conjunction with the Philips Suite App to enable a patient to record their blood pressure.
2 & 4	Philips	Health Watch[13]	A health watch that tracks heart rate and other cardio condition metrics, monitors activity, sleeping patterns, and nutrition behaviour (not a sports watch). It is best paired with a smartphone running the Philips Suite App.
3	uMotif Limited	uMotif[14]	A health app available as a mobile app, digital tool or wearable device to capture data through a graphical interface and help patients track and understand their health and symptoms. It provides a health report, connects to other wearable devices, sends medication reminders, and keeps track of regular tasks and daily activities.
4	National Museums Liverpool	House of Memories[15]	A health app that enables people living with dementia and their carers/families to keep a record of objects and experiences from the past. Patients and/or carers can create their own memory tree, memory box or memory timeline.

In summary, the LCIA Test Bed provides a structured programme of case management, monitoring, education and coaching supported by a clinical hub. It thus involves clinical and non-clinical teams, community involvement and community feedback. Good Things Foundation[16] is providing support to patients regarding their digital skills to help them use the innovator technologies.

[9] http://www.simple.uk.net
[10] http://www.cambridgecognition.com
[11] http://www.intelesant.com
[12] http://www.philips.co.uk/c-m-hs/health-programs/upper-arm-blood-pressure-monitor
[13] http://www.philips.co.uk/c-m-hs/health-programs/health-watch
[14] http://www.umotif.com
[15] http://www.liverpoolmuseums.org.uk/learning/projects/house-of-memories/
[16] http://www.goodthingsfoundation.org

For peer review only - http://bmjopen.bmj.com/site/about/guidelines.xhtml

Evaluation design

A requirement of this evaluation[17] was that it adopt a rapid cycle approach, to enable the delivery of the programme to be adapted and amended in the light of feedback received from patients and clinicians. As a result, a randomised controlled trial design was deemed unsuitable.

To address the core aims and objectives outlined above and the NHS England requirements, the evaluation was designed as a longitudinal, mixed method evaluation involving four distinct but interrelated phases to include:

1. A bespoke patient survey incorporating validated tools;
2. A mass matched control analysis;
3. Phased observational interviews with patients;
4. Weekly diaries, action learning meetings and focus groups with staff and other key stakeholders.

The different phases of the evaluation design are adopting different sampling and recruitment strategies, along with methods, as set out below. Phases 1, 3 and 4 have their own participant information sheets, consent forms and, where applicable, schedules (e.g. for the interviews and focus groups). A separate flyer has been designed for patients with dementia, providing information about the study in an informative and accessible manner, drawing on images and large font. In all instances, it will be made clear to patients that they can be supported by a carer or other family member should they wish. It is anticipated that all participants in cohort 4 (mild dementia) will be accompanied by a carer or other family member.

All materials developed for the evaluation and submitted to ethical review were shared and informed through discussion with: patient by experience groups (including those with dementia); a clinical reference group; and an evaluation advisory board. Feedback and comment from these three groups was taken into account in finalising the documents submitted for ethics approval.

1. The patient survey

Recruitment and informed consent

In line with referral to the service, recruitment is being staggered over the intervention period. The impact of the intervention on each participant is being evaluated over a six month period. Final recruitment to the evaluation will thus be at the end of month 15, to allow for the six month intervention and a three-month period for final analyses. All patients recruited into the Test Bed and receiving the service are included in the quantitative (service evaluation) stage, and are completing the Phase 1 consent form. Patients in Phase 1 are completing a baseline, mid (12 weeks) and endpoint (24 weeks) survey during the six month intervention.

Methods

The data analysis consists of a statistical evaluation of the effectiveness of the intervention and a health economic analysis of its costs and associated cost changes. The evaluation is collecting anonymised information for each participant concerning: age, gender, ethnicity, living arrangements, education completed, and use of health care services in the four weeks before completing the questionnaire such as hospital services (inpatient care, outpatient visits, and A&E visits), primary care services, social care services, and medicines. Furthermore, validated instruments to assess health related quality of life,

[17] Stipulated by NHS England

For peer review only - http://bmjopen.bmj.com/site/about/guidelines.xhtml

health and wellbeing and patient activation are being used (each of which has been validated for use with older people and can be completed by proxy if required): Warwick-Edinburgh Mental Wellbeing Scale (WEMWBS), EQ-5D-5L, Patient Activation Measure (PAM)13, and the De Jong Gierveld Loneliness Scale. These tools are reapplied at weeks 12 and 24.

The evaluation team are working with the technology innovators[18] to minimise the demands of the evaluation on patients. Participants are also being offered the option of completing the questionnaires on paper or by telephone.

Drawing on HES data, for the primary outcomes for cohorts 1 and 2 will be evaluated against a matched control group (3 controls to 1 Test Bed patient). This will be coordinated in conjunction with the Midlands and Lancashire Commissioning Support Unit.

In each cohort, the quantitative analyses will specifically address:

- Cohort 1: The mean of hospital admissions will be estimated for the Test Bed patients and for the control group. The primary outcome is the difference between the change in the two groups which will be estimated using linear regression adjusted for the matching covariates. Ninety-five percent confidence intervals will be reported for all estimated coefficients;
- Cohort 2: The evaluation is comparing compliance to healthcare treatment in the intervention group at the beginning, middle and end of the intervention;
- Cohort 3: Participants' engagement with the intervention is being assessed at the baseline, week 12 and week 24. In addition, the proportion of participants choosing to continue to use technology at the end of the intervention is being assessed;
- Cohort 4: Patient health-related quality of life is being compared at the beginning, middle and end of the intervention using various validated instruments;

The economic analysis is considering all four patient cohorts. Information on unit costs is being obtained from official sources (e.g. NHS Reference costs and Unit Costs of Health and Social Care, The British National Formulary Tariffs) and Vanguard sites.

The economic analysis is considering all four patient cohorts. Information on unit costs is being obtained from official sources (e.g. NHS Reference costs and Unit Costs of Health and Social Care, The British National Formulary Tariffs) and Vanguard sites.

2. The mass control group

The evaluation team will match 3 control subjects per intervention participant. The matching will performed using the following variables: predicted probability (risk) of hospital re-admission, long-term condition, sex, and age. The predicted probability of hospital admission for each participant and control will be calculated using the Combined Predictive Model.[9] The matching will be performed using a standard multivariate matching algorithm (Mahalanobis distance matching). The aim of matching on these variables is to obtain a control group which has similar characteristics for these variables compared to the intervention group. This should help minimise any possible confounding effects of these variables.

[18] The term 'innovators' has been coined by NHS England for the purposes of the Test Beds and refers to technology companies deemed to be working at the cutting edge of digital health design and development.

For peer review only - http://bmjopen.bmj.com/site/about/guidelines.xhtml

The statistical analysis is conducted using the statistical software Stata (version 14).

3. Phased observational interviews with patients

Recruitment and informed consent

Patient participation in Phase 2 of the evaluation is optional and participants are asked to indicate their preference for this in the Phase 1 consent form. Based on past experience, it is anticipated that more patients will indicate a willingness to take part in the second phase than will be required. Where possible, we are seeking to purposively sample on the basis of: chronic health condition(s) (cohorts 1-3); age (55-64; 65-74; 75-84; ≥85); gender; ethnicity; postal address (based on the ONS deprivation scale); and co- versus lone dwelling. Ten to fifteen patients are being recruited from each cohort to participate in the in-depth qualitative phase of the evaluation (N = 40-60). Those participating in Phase 2 are being consented at the time of their first interview.

Methods

Two observational interviews are being undertaken with each participant in their own home over the six month period of the programme: a) in the first month of receiving the intervention; and b) during the final month 6. The home-base setting is important as it facilitates a better understanding of participants' health status, health knowledge and activation prior to their participation in the programme, how they engage with the service and use the technology within their own homes at the outset and whether this changes over time. This phase is specifically designed to gain a better understanding of: how participants engage with and experience the technology and Fylde Coast Vanguard / Better Care Together services; how this may influence patient activation and self-management of care; any barriers to improving self-care; whether the intervention can increase their sense of empowerment and independence; service satisfaction; and overall quality of life.

4. Understanding the impact on staff and key stakeholders: diaries, action learning meetings and focus groups

In the early stage of the Test Bed, focus groups will be conducted with key stakeholders to determine the lessons learned in the process of operationalising the Test Bed. This data will begin to inform and help map out the logic model (see below). Informed consent will be taken for all participants.

All key stakeholders and service providers involved in the delivery of the service are also invited to complete brief weekly diaries and participate in regular 'action learning' meetings, informed by the diary data, as part of a rapid cycle review process. Here, members of the evaluation team meet regularly with key stakeholders involved in the implementation and delivery of the service to reflect on shared learning, agreed action and the impacts of change. This includes technology innovators, members of staff from the Test Bed hubs, voluntary sector providers and others. Numbers for these meetings are anticipated to be around 20 people, but should numbers increase beyond this we will ask stakeholders to nominate representatives to participate in the meetings and act as two-way conduits for the learning from their particular stakeholder group.

Action learning meetings will take place at three-monthly intervals and will involve a cycle of discussion, shared learning and reflection and agreed action to be taken forward regarding the operation, delivery and effectiveness of the service. Agreed action from these meetings will build on this cycle of learning and assessment of the service throughout the Test Bed period. To facilitate discussion at these meetings, key stakeholders are asked to complete weekly diaries documenting their experiences of the service, including what works well and less well, and any observations of patients' responses to the

For peer review only - http://bmjopen.bmj.com/site/about/guidelines.xhtml

service (time spent/support need/technical difficulties). Diaries are brief and simple to complete and are designed to feed into the action research meetings and to fine-tune themes for the focus groups and deliberative panels. The diaries are submitted electronically with participants being encouraged to keep a personal copy as an 'aide memoire' prior to each action learning meeting.

Toward the end of the evaluation we will also hold eight focus groups (N=6-10 participants per focus group) with key stakeholders involved in the delivery of the service. Our focus here is on recruiting not only those directly involved in delivering the service to older participants but also those organising and managing it. Recruitment to the focus groups will be split evenly across the two sites. The aim here will be to gain an in-depth understanding of how Fylde Coast Vanguard/Better Care Together services with the technology is impacting on working practices. The focus groups will also explore the extent to which the programme may help to increase: communication between the care teams; productivity; capacity and co-ordination of care; and overall work satisfaction.

For ease of reference, a summary of the overall evaluation methods is set out in Table 4 below.

Table 4: Summary of overall evaluation methods

Participants	Survey	Observational interview	Focus groups	Diaries	Action Learning meetings	Deliberative panels
Patients (with carers as appropriate)	Yes (3 over 6 months)	Yes (2 over 6 months)	X	X	X	A sample
Staff delivering service	X	X	Yes	Yes	Yes	A sample
Other key stakeholders (technology innovators, voluntary sector etc)	X	X	Yes	X	Yes	A sample
Managers/administrators	X	X	Yes	X	X	A sample

ETHICS AND DISSEMINATION

Audio recording

All qualitative data is being audio recorded using an encrypting digital voice recorder. In the process of transcription, names of participants are anonymized and any identifying features removed or coded. These points are included in the consent forms and are discussed with each participant before the interview/action research meeting/focus group/deliberative panel. All audio files will be deleted from the recorder once data analysis is complete.

Data analysis

The qualitative data will be analysed thematically with the aid of Atlas.ti qualitative software. Initial identification of emerging themes and initial coding will be undertaken by the field researcher then shared and agreed during evaluation team data analysis workshops. This will allow for the verification of the coding framework and enable the evaluation team to develop an analysis of the situation and understand the phenomenon being explored.[10] The major codes emerging from the data will be presented to the deliberative panels for fine-tuning and final verification.

Appendix 1 contains information regarding participant payment, data storage, participant benefit, potential risk to participants, withdrawal and researcher risk.

For peer review only - http://bmjopen.bmj.com/site/about/guidelines.xhtml

Deliberative panels

In the final three months of the evaluation, we will hold two deliberative panels (one per Vanguard site: N=15-20 per panel) with key stakeholders to include: older participants; members of the Vanguard care teams; key stakeholders from clinical and community settings, and social care; innovators; and senior managers and commissioners from the Test Bed sites.

Deliberative panels are designed to validate and fine-tune outcomes by producing an informed and collective view resulting from deliberation. They offer a bottom-up approach that can shift evaluation findings into outcomes related to policy, practice and guidance for employers. To achieve this, draft findings emerging from the data analyses will be presented with the aim of drawing on the various expertise of the participants.

In this case, it will help to identify and validate those key components of the intervention that will comprise a new model of care that may be scaled up for wider roll-out and will help to finalise the evaluation recommendations. The deliberative panels will also be used to begin preliminary discussions of how this model might form the basis of a joint health and social care strategy for telehealth/telecare. Finally, the panels will help us to fine-tune the Logic Model through a process of 'backward mapping' (see below).

The Logic Model framework

The evaluation is located within a Logic Model framework, enabling us to understand the impact of the intervention. It also enables us to consider the processes, management and participation that may have implications for the Test Bed's success. In developing the Logic Model, we are considering the following key elements:

- Activities – the focus of our model is the New Models of Care with Technology intervention. The programme of work is considered in its context, including the partners of the project and the policies and procedures that are in place.
- Inputs – including the financial, human, organisational, and material resources of the project. This includes the following stakeholders: older adults taking part in the project; Fylde Coast Vanguard and Better Care Together teams; clinicians; GPs; community nurses; other health professionals; and innovator, voluntary and community sector input.
- Outcomes – the desired and actual results of the project and a Logic Model that will support roll out and scalability.

The Logic Model has been designed as a processual model that will be populated using data gathered in the diaries and action research meetings. The elements identified above will be fine-tuned iteratively over the two years of the intervention and finalised through a process of 'backward mapping' during the final deliberative panels. In this way, the final Logic Model, designed to assist with wider roll-out of the intervention, will be developed by drawing on informed and collective views resulting from ongoing iteration and deliberation involving all key stakeholders.

Dissemination

The findings of the evaluation will be disseminated in the following ways:

For peer review only - http://bmjopen.bmj.com/site/about/guidelines.xhtml

a) A final evaluation report which also contributes to the national evaluation;
b) Presentations to all key stakeholders within the Vanguard sites and NHS England;
c) Conference papers and posters presented at regional, national, and international conferences;
d) Publication in a range of high quality, international, peer-reviewed scientific journals;
e) Publication in a range of journals/publications relevant to service providers;
f) Dissemination of evaluation highlights through social media.

FULL REFERENCES

1 George J & Martin F (2016) Briefing paper: Living with long-term conditions. British Medical Association. Available at: https://www.bma.org.uk/-/media/files/pdfs/.../living-with-long-term-conditions.pdf [Accessed on 6 April 2017].

2 King's Fund, The (2016) Quarterly Monitoring Report, No. 21. Available at: http://qmr.kingsfund.org.uk/2016/21?_ga=1.123564811.1874139399.1487344226/ (November 2016) [Accessed on 6 April 2017].

3 Local Government Association, The (2016) 'Adult Social Care Funding: 2016 State of the Nation Report'. November 2016. Available at: http://www.local.gov.uk/documents/10180/7632544/1+24+ASCF+state+of+the+nation+2016_WEB.pdf/e5943f2d-4dbd-41a8-b73e-da0c7209ec12 [Accessed on 6 April 2017].

4 NHS Blackpool Clinical Commissioning Group (2017) 'Extensive Care'. Available at: http://blackpoolccg.nhs.uk/local-services/new-models-of-care/extensive-care/ [Accessed on 6 April 2017].

5 Bay Health and Care Partners (2017) 'Guide to Better Care Together'. Available at: http://clinicalimages.web.furness.net/files/BCT-Guide-13Jul.pdf [Accessed on 6 April 2017].

6 NHS England (2017) 'About vanguards'. Available at: https://www.england.nhs.uk/ourwork/new-care-models/vanguards/about-vanguards/ [Accessed on 6 April 2017].

7 van Berkel C, Smith M, Horsfield D. et al. Evidence for supported self-care at scale: A population approach to evaluating technology enabled support for long term condition management. Liverpool, UK: Philips and Liverpool Clinical Commissioning Group 2016.

8 van Berkel C, Smith M, Horsfield D. et al. Evidence for supported self-care at scale. *International Journal of Integrated Care* 2016 16(5) DOI: dx.doi.org/10.5334/ijic..2595

9 Wennberg D, Siegel M, Darin B, et al. Combined Predictive Model: Final report and technical documentation. London, UK: The King's Fund 2006.

10 Bowling A. Research Methods in Health: Investigating Health and Health Services, Fourth Edition. Maidenhead, UK: Open University Press 2014.

11 Data Protection Act (1998) Data Protection Act. Available at: http://www.legislation.gov.uk/UKPGA/1998/29/contents [Accessed on 17 February 2017].

For peer review only - http://bmjopen.bmj.com/site/about/guidelines.xhtml

12 Bryman A. Social Research Methods, Sixth Edition. Oxford, UK: Oxford University Press 2016.

AUTHORS' CONTRIBUTIONS

Sandra Varey is the principal author and wrote the majority of the manuscript. Alejandra Hernández, Thomas Palmer, Céu Mateus, Joann Wilkinson, Mandy Dixon and Christine Milligan are co-authors and each contributed to the editing and revision of the manuscript through several iterations. Christine Milligan is the Principal Investigator for the evaluation and is the corresponding author.

FUNDING STATEMENT

This work is supported by NHS England.

COMPETING INTERESTS STATEMENT

The authors declared no potential conflicts of interest with respect to the research, authorship, and/or publication of this article.

Acknowledgements

The authors wish to thank Amanda Thornton, the Patient by Experience groups, the Clinical Reference group and the LCIA Test Bed Evaluation Advisory Board for their helpful feedback on the initial design of the protocol materials.

In the original bid documentation, Philips contributed to the development of Table 2 presented in this paper.

For peer review only

For peer review only - http://bmjopen.bmj.com/site/about/guidelines.xhtml

Appendix 1

Participant payment

Participants taking part in the qualitative elements of the study will receive a voucher of £20 as a thank you in recognition of the time they have given to taking part in the evaluation. In the quantitative elements of the evaluation we are unable to offer any incentive because of the large numbers of participants.

Data storage

Personal data is being stored on a password safe university computer accessible only to the researchers. The research data is stored in an encrypted file using the software TrueCrypt.

All data is being stored securely in line with the Data Protection Act [11] and will be kept no longer than necessary.[12] Written data will be kept for a maximum of 10 years in a secure cabinet. Any electronic copies of data will be kept on the University server, which is a password protected system and will be encrypted. If electronic data requires transfer, this is done on an encrypted USB device. Any emails sent from participants (e.g. electronic diaries) are sent to a password secured email address that has been set up for the purpose of the study. Emails are saved electronically with any other electronic copies of data, with the email then being deleted. Where emails and interview/focus group data are printed for the purposes of analysis, they are stored with other written data in a secure cabinet. At the end of the study, the email account will be deleted. The paper copies of any participant contact details will be scanned and stored electronically, and paper copies destroyed.

Participant benefit

Participants have the direct benefit of using the technology during the two years of the Test Bed; however the Test Bed team recognise the need to make clear for those in Cohorts 1 and 4 that this is time-limited. Patients (particularly those in cohorts 2 and 3) for whom the intervention is a text messaging system or an app, will have access to these beyond the six month timeframe of the Test Bed programme. Given a key objective of the service is to enhance patient activation and ability to self-care, it is also anticipated that potential ongoing benefits will support older people with chronic health problems to remain in their own homes longer.

Patients and health professionals also have an opportunity to share directly with researchers their experiences of using and implementing this model of care. In this way, all participants can take an active part in shaping current and future healthcare services in place for older people with chronic conditions or dementia who are living at home.

Potential risk to participants

For patients participating in Phase 1 of the evaluation, there are no anticipated risks from participation in the project. All validated tools are validated for completion by proxy, and patients and family

For peer review only

members are therefore made aware that the patient can be supported to complete the surveys if required.

For patients taking part in Phase 2 of the evaluation research, no specific risks are anticipated. However, the following considerations have been made:

1. It is possible that a patient may become distressed by the presence of the researcher in their home during the observational interviews. To avoid this risk, the researcher will take care to check with the patient at the beginning of the interview to ensure they are happy to continue taking part in the study. The researcher will also do this at the end of the session and for the second interview.

2. If a patient should become distressed for any reason during an observational interview, the researcher will provide the patient with the contact details of different support networks for older people, as outlined in the participant information sheet. A member of the Test Bed hub will also be alerted should this be necessary, in order to assess whether a visit from a member of the hub is required.

Participants involved in the action learning meetings and the focus groups will be reminded that discussion in these settings is confidential and information shared (other than the shared and agreed action learning) should not be discussed with others outside of the meeting.

Withdrawal

Participants may choose to leave the study at any time and are assured that their data can be withdrawn up to four weeks after collection. The rationale for this four week time period is that, beyond four weeks, analysis will have begun. Those participating in focus groups are advised that, although every effort will be undertaken to remove their data should they request it during the timeframe, this may not always be possible. This is due to difficulties in identifying/attributing specific comments to individuals and because focus group data is often the outcome of group discussion, making it hard to distil to whom individual thoughts/comments should be attributed.

Researcher risk

There is limited risk to the quantitative researchers as they have very limited direct engagement with the participants (survey completion is largely through technology or telephone).

The qualitative researcher is interviewing participants in their own homes and is experienced in undertaking this type of fieldwork. The researcher is adhering to the University's lone worker policy. In order to minimise any potential risks, for each home visit conducted, the researcher identifies a 'buddy' (a member of staff within the University) with whom to remain in contact with: before the interview commences; when the interview has been terminated; and when the researcher has left the patient's home. The buddy is informed of the time and place of the interview, along with a contact telephone number.

Researchers within the team have the opportunity to debrief with other members of the team if required and have full access to counselling services if necessary.

For peer review only

BMJ Open

How effective and cost effective are innovative combinatorial technologies and practices for supporting older people with long-term conditions to remain well in the community? An evaluation protocol for an NHS Test Bed in Northwest England.

For peer review only

Journal:	*BMJ Open*
Manuscript ID	bmjopen-2017-017268.R1
Article Type:	Protocol
Date Submitted by the Author:	24-Aug-2017
Complete List of Authors:	Varey, Sandra; Lancaster University, Division of Health Research<br>Hernândez, Alejandra; Lancaster University<br>Palmer, Tom; Lancaster University, Department of Mathematics and Statistics ; University of Warwick, Warwick Medical School, Division of Health Sciences<br>Mateus, Ceu; University of Lancaster, Division of Health Research<br>Wilkinson, Joann; Lancaster University<br>Dixon, Mandy; Lancaster University<br>Milligan, Christine; Lancaster University
**Primary Subject Heading**:	Health services research
Secondary Subject Heading:	Geriatric medicine
Keywords:	Health technologies, older people, long-term conditions, patient activation, self-care

SCHOLARONE™
Manuscripts

Title: How effective and cost effective are innovative combinatorial technologies and practices for supporting older people with long-term conditions to remain well in the community? An evaluation protocol for an NHS Test Bed in Northwest England.

Sandra Varey[1], Alejandra Hernández[1], Tom Palmer[2], Céu Mateus[3], Joann Wilkinson[1], Mandy Dixon[4] and Christine Milligan[1]

1. Centre for Ageing Research, Division of Health Research, Lancaster University, Lancaster LA1 4YG, United Kingdom

2. Department of Mathematics and Statistics, Lancaster University, Lancaster LA1 4YG, United Kingdom

3. Health Economics, Division of Health Research, Lancaster University, Lancaster LA1 4YG, United Kingdom

4. Lancaster Health Hub, Lancaster University, Lancaster LA1 4YG, United Kingdom

Correspondence to:

Professor Christine Milligan

Centre for Ageing Research

Division of Health Research

Lancaster University

Lancaster LA1 4YG

United Kingdom

Email: c.milligan@lancaster.ac.uk

Telephone: +44 1524 592128

Word count: 4261

For peer review only - http://bmjopen.bmj.com/site/about/guidelines.xhtml

Abstract

Introduction:
The Lancashire and Cumbria Innovation Alliance (LCIA) Test Bed is a partnership between the UK National Health Service (NHS), industry (led by Philips) and Lancaster University. Through the implementation of a combination of innovative health technologies and practices, it aims to determine the most effective and cost effective ways of supporting frail older people with long-term conditions to remain well in the community. Among the Test Bed's objectives are to: improve patient activation and the ability of older people to self-care at home; reduce healthcare system utilisation; and deliver increased workforce productivity.

Methods and Analysis:
Patients aged 55 years and over are recruited to four cohorts defined by their risk of hospital admission, with long-term conditions including COPD, dementia, diabetes and heart failure. Interventions are determined on an individual basis, with a range of technologies available. The evaluation is adopting a two-phase approach: Phase 1 includes a bespoke patient survey and a mass matched control analysis; and Phase 2 is using observational interviews with patients, and weekly diaries, action learning meetings and focus groups with members of staff and other key stakeholders. Phase 1 data analysis consists of a statistical evaluation of the effectiveness of the intervention and a health economic analysis of its costs and associated cost changes. Phase 2 data will be analysed thematically with the aid of Atlas.ti qualitative software. The evaluation is located within a logic model framework, to consider the processes, management and participation that may have implications for the Test Bed's success.

Ethics and Dissemination:
The LCIA Test Bed evaluation has received ethical approval from the Health Research Authority and Lancaster University's Faculty of Health and Medicine Research Ethics Committee. A range of dissemination methods are adopted including deliberative panels with key stakeholders to validate findings and develop outcomes for policy and practice.

Word count: 300

Strengths and limitations of this study

- The evaluation is considering both the effectiveness, in terms of measures such as hospital admissions, and cost effectiveness of the Test Bed
- The protocol evaluates the potential for patient activation through health technology
- Observational interviews with patients in their own homes enable insights into how older people engage with the technology
- For both the Test Bed participants and controls the evaluation has to use proxy measures of the costs incurred to the NHS
- Due to the timescale of the overall programme, it is not possible to assess the longer-term impact post-intervention

For peer review only - http://bmjopen.bmj.com/site/about/guidelines.xhtml

Key words

Health technologies; older people; long-term conditions; patient activation; self-care.

INTRODUCTION

In the UK, increasing numbers of older people are living with long-term conditions including diabetes, chronic obstructive pulmonary disease (COPD), heart failure and dementia.[1] This is contributing to increased pressure on health and social care systems including accident and emergency services, which in turn increases demand on the number of hospital beds.[2] At the same time, local authorities are experiencing a reduction in the funding available to provide social care.[3]

Concerns about this so-called care-gap and our future ability to cope with the growing numbers of older people with long-term conditions underpins governmental drivers to develop new models of care that are cost-effective and which facilitate older people's ability to better self-manage their care needs at home. The NHS England Test Bed programme has thus been designed to encourage the trialling of new models of care that are supported by health technologies across a number of areas in the UK.

This paper reports on the protocol for the evaluation of one such programme, the Lancashire and Cumbria Innovation Alliance (LCIA) Test Bed in the north-west of England. The programme commenced on 1st April 2016 and is due to be completed on 31st March 2018.

The LCIA Test Bed initiative

The LCIA Test Bed[1] is a partnership between the NHS, industry (led by Philips) and Lancaster University, and is one of seven Test Beds located across England. Two neighbouring Vanguard sites[2], part of the Lancaster Health Hub (an established NHS/University partnership comprising ten local organisations), will deliver the Test Bed. The Test Bed's aims and objectives are presented below.

Test Bed Aims:
To determine the most effective and cost effective ways of supporting frail older people with dementia and other long-term conditions (LTCs) to remain well in the community and avoid unnecessary hospital admissions.

Test Bed Objectives:
To use a combinatorial range of technologies and interventions to:

- better support frail older people, older people living with long-term conditions;
- improve patient activation and the ability of older people with a range of LTCs to self-care at home;
- improve health awareness and outcomes for older people with LTCs;
- reduce healthcare system utilisation and deliver increased workforce productivity.

[1] For more information, visit: https://www.england.nhs.uk/ourwork/innovation/test-beds/innovation-alliance/

[2] In 2015, the National Health Service in England selected 50 vanguard sites to take a lead on the development of new integrated care models designed to act as blueprints for the future of the NHS. Complete redesign of whole health and care systems are being considered. The hope is that the vanguards will provide inspiration to the rest of the health and care system across the UK (NHS England, 2017).[4]

For peer review only - http://bmjopen.bmj.com/site/about/guidelines.xhtml

Over two years, the Test Bed is implementing a combination of innovative technologies and practices aimed at supporting these aims and objectives. The clinical challenge is to reduce hospital admissions, create capacity, reduce overall costs, minimize other health and social care usage, and maximize patient outcomes for a targeted population of older people (defined in this Test Bed as those aged 55 years and over) who suffer a range of long term conditions including dementia. These LTCs present a major challenge for the area given its dispersed population, and innovative solutions are urgently required.

The main technology partner is Philips who, together with a number of small and medium sized enterprises (SMEs), social enterprises, and voluntary organisations, is working with the Test Bed to introduce a health technology-enabled supported self-care programme[3].

The new model of care includes services in the two Vanguard sites in the Fylde Coast and North Lancashire. The Fylde Coast Vanguard[4] is using a range of health services; one of these is Extensive Care, which brings together a range of services in the same location to achieve a co-ordinated team of healthcare professionals, with patients each allocated a wellbeing support worker.[5] In the North Lancashire Vanguard, Better Care Together sees healthcare professionals working together in partnership to help people manage their own health conditions.[6] As part of the LCIA Test Bed programme, these existing services are being combined with innovative technologies and support for older people with LTCs including COPD, Heart Failure, Diabetes and Dementia. For those with lower levels of risk in cohort 3 (see Table 1), asthma and hypertension will also be included. The model of care in this Test Bed is designed to empower patients to actively manage their health conditions and change behaviour through the provision of appropriate coaching and education.

A core part of the programme also focuses on increasing involvement of the third sector and community organisations in providing support for this new model of care with technology; and understanding the impact of the intervention on staff and key stakeholders.

Core outcomes for the evaluation

The Test Bed focuses on a combinational approach to technologies. Hence, rather than focusing on the impact of any single technology, the Test Bed evaluation is focusing on how health technologies as an addition to existing Vanguard services can improve and promote self-care at home and reduce hospital admissions for older people with LTCs.

The evaluation is focusing on three primary outcomes:

1. The extent to which supported self-care telehealth technology can improve patient outcomes, through reduced hospital admissions, medication, GP visits, home visits and community service use;

2. The extent to which supported self-care telehealth technology can improve patient activation for frail older people with LTCs (to include increased sense of empowerment, improved quality of life, service satisfaction, enhanced knowledge, skills and confidence to self-manage care);

[3] Cambridge Cognition Limited; Good Things Foundation; Intelesant Limited; MKS Solutions Limited; Simple Shared Healthcare Limited; and uMotif Limited.

[4] The Fylde Coast Vanguard encompasses NHS Blackpool CCG, NHS Fylde & Wyre CCG and Blackpool Teaching Hospitals.

For peer review only - http://bmjopen.bmj.com/site/about/guidelines.xhtml

3. The cost effectiveness of the intervention and how it might be scaled up to provide better value for patients and taxpayers.

Secondary outcomes include:

1. Identification of the strengths and weaknesses of the intervention and how these may be built on or addressed;

2. Assessment of patient/staff perceptions of how the intervention improves on existing services and makes best use of voluntary and community services;

3. Assessment of the impact of the intervention on the workforce in terms of communication between care teams, productivity, capacity, co-ordination of care, and work satisfaction.

To achieve this, we are:

a) Using quantitative and qualitative measures to examine benefits to patients and patient outcomes as identified above;

b) Undertaking an economic evaluation of the impact of adding a combination of technologies to support and promote self-care in the provision of healthcare in comparison to standard care;

c) Drawing on a Logic Model to demonstrate how to construct the service as a scalable model that might provide better value to patients and taxpayers.

METHODS AND ANALYSIS

Ethical approvals

The evaluation is adopting a two-phase approach: Phase 1 focuses on service evaluation and is quantitative in its design; while Phase 2 focuses on more in-depth understandings of patient and staff experiences of the service through qualitative research.

These two distinct stages have received ethical approval through two different routes: Phase 1 was deemed by the Health Research Authority (HRA) to be service evaluation not requiring NHS ethics approval. However, it was submitted and has been awarded ethical approval from Lancaster University's Faculty of Health and Medicine Research Ethics Committee (Reference: FHMREC16025). Phase 2 was deemed by HRA to be research and thus was submitted and has received ethical approval from the HRA (IRAS Project ID: 208395). Further details about the two phases are provided below.

Phase 2 of the evaluation has been adopted onto the National Institute for Health Research portfolio.[5]

Sampling and recruitment

[5] NIHR Portfolio reference number is the same as the IRAS number (IRAS Project ID 208395)

For peer review only - http://bmjopen.bmj.com/site/about/guidelines.xhtml

Patients are being recruited to four cohorts defined by their risk of hospital admission. The long-term conditions are noted above and are outlined in Table 1.

Table 1: Recruitment criteria for the four LCIA Test Bed cohorts

Cohort	Age	Risk of hospital admission	Long-term conditions
Cohort 1	Aged 55 years or over	>25%	COPD, HF
Cohort 2		>10% and <25%	
Cohort 3		<10%	Diabetes, Asthma, CHD, Hypertension
Cohort 4		N/A	Early stage dementia (ACE-III assessment tool)

Participants for cohorts 1, 2 and 3 are being recruited to the Test Bed through the Vanguard services to which they are already assigned. As illustrated in Table 1, Cohort 4 is focusing specifically on patients with early stage dementia, as identified through the ACE-III assessment tool. Potential participants for cohort 4 are being recruited through the Memory Assessment Service (MAS) in the Fylde Coast and North Lancashire. Those who have received a recent diagnosis of mild dementia by a consultant psychiatrist following their comprehensive assessment with the MAS are deemed as having fulfilled the entry criteria of mild dementia. For these patients, the ACE-III or Mini-ACE has been completed as part of their cognitive assessment at the MAS clinic, the results of which have aided the formulation of the dementia diagnosis.

All patients consenting to the take part in the Test Bed and to receiving the intervention are required to consent to participate in Phase 1 of the service evaluation. Participation in the qualitative Phase 2 of the evaluation is optional. Patients indicating an interest in participating in Phase 2 are being consented separately through a two-phase consent process.

The sample size for recruitment to each cohort has been informed by population and risk data for the area, along with the methodology used in a previous study.[7,8] As Table 2 illustrates, the overall aim of the Test Bed is to recruit 1600 patients; however, given the anticipated challenges of recruitment and potential attrition due to the nature of the patient group, we estimate an overall recruitment and retention figure of 60% (overall N=960).

Table 2: LCIA Test Bed cohort sample sizes (*combined over a population of app. 310,000 people)[6]

[6] See Acknowledgements

For peer review only - http://bmjopen.bmj.com/site/about/guidelines.xhtml

Cohort	Percent of the total population*	Total patients	Percent of frail and elderly patients	Percent of patients with dementia	Number of patients estimated to be appropriate for the service	Percent of appropriate patients within cohort that will be recruited within the Test Bed period	Minimum number of patients recruited within Test Bed period	Estimated number of patients by the end of the Test Bed period
**1** (age>55, risk>25% & 1 LTC)	1.70%	5,262	20%	3.5%	1,737	30%	521	500
**2** (age>55, 10%>risk<25 % & 1 LTC)	3.00%	9,286	4%	1.1%	3,064	15%	460	500
**3** (age>55, 10%<risk 1 of a broader set of LTCs)	9.20%	28,477	2%	1.6%	9,398	5%	470	500
**4** (diagnosis of dementia)	0.79%	2,430		100.0%	810	10%	81	100

Allowing for a phased increase in recruitment over the intervention period, the overall recruitment is estimated at approximately 60-70% of the figures identified in Table 2. This will provide sufficient data from cohorts 1 and 2 to demonstrate a change in hospital admissions (a primary outcome). It is unlikely that the evaluation will be able to demonstrate statistical significance for cohorts 3 and 4 for this outcome, although other measures of wellbeing, patient activation, and effectiveness of the technologies will be assessed. The secondary outcomes are being supported by observational interviews across all four cohorts.

The intervention

The patient interface varies across the four cohorts, and the technologies available to people within the different cohorts are presented in Table 3. A patient's intervention is determined on an individual basis and is informed by a number of factors, including health condition(s), personal preferences and other needs. Some patients are using different technologies at different periods of time within the six month intervention.

Table 3: Technologies available to each cohort

Cohort	Company	Technology	Description
1 & 4	Philips	Motiva[7]	A telehealth platform which operates through a tablet or TV set top box interface that connects wirelessly to a range of tele-monitoring equipment in the home (e.g. wireless weighing scales, blood pressure meters, pulse oximeters and thermometers).
	MKS Solutions Limited	Speakset[8]	A video calling system that connects a health professional with a patient through the patient's TV in their own home.

[7] http://www.philips.co.uk/healthcare/solutions/enterprise-telehealth/home-telehealth
[8] http://www.speakset.com

For peer review only - http://bmjopen.bmj.com/site/about/guidelines.xhtml

	NHS Stoke-on-Trent Clinical Commissioning Group's Licence	Florence/NHS Simple[9]	A short message communication software providing a reminder/communication text messaging service for patients.
1, 2 & 3	Cambridge Cognition Limited	CANTAB Mobile[10]	An assessment tool designed for healthcare professionals to identify the earliest signs of clinically significant memory impairment. The assessment comprises three tests: the Paired Associates Learning (PAL) test to assess episodic memory; the Geriatric Depression Scale (GDS) to identify signs of depression and an Activities of Daily Living questionnaire (ADL) to assess functionality in daily life.
2	Intelesant Limited	Intelesant[11]	A mobile app to support daily management of COPD through SMS text messaging alerts for coaching, prompts, and reminders. It incorporates the individual's action plan in response to changes in symptoms.
	Philips	Personal Blood Pressure Cuff[12]	A blood pressure monitor that works in conjunction with the Philips Suite App to enable a patient to record their blood pressure.
2 & 4	Philips	Health Watch[13]	A health watch that tracks heart rate and other cardio condition metrics, monitors activity, sleeping patterns, and nutrition behaviour (not a sports watch). It is best paired with a smartphone running the Philips Suite App.
3	uMotif Limited	uMotif[14]	A health app available as a mobile app, digital tool or wearable device to capture data through a graphical interface and help patients track and understand their health and symptoms. It provides a health report, connects to other wearable devices, sends medication reminders, and keeps track of regular tasks and daily activities.
4	National Museums Liverpool	House of Memories[15]	A health app that enables people living with dementia and their carers/families to keep a record of objects and experiences from the past. Patients and/or carers can create their own memory tree, memory box or memory timeline.

In summary, the LCIA Test Bed provides a structured programme of case management, monitoring, education and coaching supported by a clinical hub. It thus involves clinical and non-clinical teams, community involvement and community feedback. Good Things Foundation[16] is providing support to patients regarding their digital skills to help them use the innovator technologies.

[9] http://www.simple.uk.net
[10] http://www.cambridgecognition.com
[11] http://www.intelesant.com
[12] http://www.philips.co.uk/c-m-hs/health-programs/upper-arm-blood-pressure-monitor
[13] http://www.philips.co.uk/c-m-hs/health-programs/health-watch
[14] http://www.umotif.com
[15] http://www.liverpoolmuseums.org.uk/learning/projects/house-of-memories/
[16] http://www.goodthingsfoundation.org

For peer review only - http://bmjopen.bmj.com/site/about/guidelines.xhtml

Evaluation design

A requirement of this evaluation[17] was that it adopt a rapid cycle approach, to enable the delivery of the programme to be adapted and amended in the light of feedback received from patients and clinicians. As a result, a randomised controlled trial design was deemed unsuitable.

To address the core aims and objectives outlined above and the NHS England requirements, the evaluation was designed as a longitudinal, mixed method evaluation involving four distinct but interrelated phases to include:

1. A bespoke patient survey incorporating validated tools;
2. A mass matched control analysis;
3. Phased observational interviews with patients;
4. Weekly diaries, action learning meetings and focus groups with staff and other key stakeholders.

The different phases of the evaluation design are adopting different sampling and recruitment strategies, along with methods, as set out below. Phases 1, 3 and 4 have their own participant information sheets, consent forms and, where applicable, schedules (e.g. for the interviews and focus groups). A separate flyer has been designed for patients with dementia, providing information about the study in an informative and accessible manner, drawing on images and large font. In all instances, it will be made clear to patients that they can be supported by a carer or other family member should they wish. It is anticipated that all participants in cohort 4 (mild dementia) will be accompanied by a carer or other family member.

All materials developed for the evaluation and submitted to ethical review were shared and informed through discussion with: patient by experience groups (including those with dementia); a clinical reference group; and an evaluation advisory board. Feedback and comment from these three groups was taken into account in finalising the documents submitted for ethics approval.

1. The patient survey

Recruitment and informed consent

In line with referral to the service, recruitment is being staggered over the intervention period. The impact of the intervention on each participant is being evaluated over a six month period. Final recruitment to the evaluation will thus be at the end of month 15, to allow for the six month intervention and a three-month period for final analyses. All patients recruited into the Test Bed and receiving the service are included in the quantitative (service evaluation) stage, and are completing the Phase 1 consent form. Patients in Phase 1 are completing a baseline, mid (12 weeks) and endpoint (24 weeks) survey during the six month intervention.

Methods

The data analysis consists of a statistical evaluation of the effectiveness of the intervention and a health economic analysis of its costs and associated cost changes. The evaluation is collecting anonymised information for each participant concerning: age, gender, ethnicity, living arrangements, education completed, and use of health care services in the four weeks before completing the questionnaire such as hospital services (inpatient care, outpatient visits, and A&E visits), primary care services, social care services, and medicines. Furthermore, validated instruments to assess health related quality of life,

---

[17] Stipulated by NHS England

For peer review only - http://bmjopen.bmj.com/site/about/guidelines.xhtml

health and wellbeing and patient activation are being used (each of which has been validated for use with older people and can be completed by proxy if required): Warwick-Edinburgh Mental Wellbeing Scale (WEMWBS), EQ-5D-5L, Patient Activation Measure (PAM)13, and the De Jong Gierveld Loneliness Scale. These tools are reapplied at weeks 12 and 24.

The evaluation team are working with the technology innovators[18] to minimise the demands of the evaluation on patients. Participants are also being offered the option of completing the questionnaires on paper or by telephone.

Drawing on HES data, for the primary outcomes for cohorts 1 and 2 will be evaluated against a matched control group (3 controls to 1 Test Bed patient). This will be coordinated in conjunction with the Midlands and Lancashire Commissioning Support Unit.

In each cohort, the quantitative analyses will specifically address:

- Cohort 1: The mean of hospital admissions will be estimated for the Test Bed patients and for the control group. The primary outcome is the difference between the change in the two groups which will be estimated using linear regression adjusted for the matching covariates. Ninety-five percent confidence intervals will be reported for all estimated coefficients;
- Cohort 2: The evaluation is comparing compliance to healthcare treatment in the intervention group at the beginning, middle and end of the intervention;
- Cohort 3: Participants' engagement with the intervention is being assessed at the baseline, week 12 and week 24. In addition, the proportion of participants choosing to continue to use technology at the end of the intervention is being assessed;
- Cohort 4: Patient health-related quality of life is being compared at the beginning, middle and end of the intervention using various validated instruments;

The economic analysis is considering all four patient cohorts. Information on unit costs is being obtained from official sources (e.g. NHS Reference costs and Unit Costs of Health and Social Care, The British National Formulary Tariffs) and Vanguard sites.

The economic analysis is considering all four patient cohorts. Information on unit costs is being obtained from official sources (e.g. NHS Reference costs and Unit Costs of Health and Social Care, The British National Formulary Tariffs) and Vanguard sites.

2. The mass control group

The evaluation team will match 3 control subjects per intervention participant. The matching will performed using the following variables: predicted probability (risk) of hospital re-admission, long-term condition, sex, and age. The predicted probability of hospital admission for each participant and control will be calculated using the Combined Predictive Model.[9] The matching will be performed using a standard multivariate matching algorithm (Mahalanobis distance matching). The aim of matching on these variables is to obtain a control group which has similar characteristics for these variables compared to the intervention group. This should help minimise any possible confounding effects of these variables.

[18] The term 'innovators' has been coined by NHS England for the purposes of the Test Beds and refers to technology companies deemed to be working at the cutting edge of digital health design and development.

For peer review only - http://bmjopen.bmj.com/site/about/guidelines.xhtml

The statistical analysis is conducted using the statistical software Stata (version 14).

3. Phased observational interviews with patients

Recruitment and informed consent

Patient participation in Phase 2 of the evaluation is optional and participants are asked to indicate their preference for this in the Phase 1 consent form. Based on past experience, it is anticipated that more patients will indicate a willingness to take part in the second phase than will be required. Where possible, we are seeking to purposively sample on the basis of: chronic health condition(s) (cohorts 1-3); age (55-64; 65-74; 75-84; ≥85); gender; ethnicity; postal address (based on the ONS deprivation scale); and co- versus lone dwelling. Ten to fifteen patients are being recruited from each cohort to participate in the in-depth qualitative phase of the evaluation (N = 40-60). Those participating in Phase 2 are being consented at the time of their first interview.

Methods

Two observational interviews are being undertaken with each participant in their own home over the six month period of the programme: a) in the first month of receiving the intervention; and b) during the final month 6. The home-base setting is important as it facilitates a better understanding of participants' health status, health knowledge and activation prior to their participation in the programme, how they engage with the service and use the technology within their own homes at the outset and whether this changes over time. This phase is specifically designed to gain a better understanding of: how participants engage with and experience the technology and Fylde Coast Vanguard / Better Care Together services; how this may influence patient activation and self-management of care; any barriers to improving self-care; whether the intervention can increase their sense of empowerment and independence; service satisfaction; and overall quality of life.

4. Understanding the impact on staff and key stakeholders: diaries, action learning meetings and focus groups

In the early stage of the Test Bed, focus groups will be conducted with key stakeholders to determine the lessons learned in the process of operationalising the Test Bed. This data will begin to inform and help map out the logic model (see below). Informed consent will be taken for all participants.

All key stakeholders and service providers involved in the delivery of the service are also invited to complete brief weekly diaries and participate in regular 'action learning' meetings, informed by the diary data, as part of a rapid cycle review process. Here, members of the evaluation team meet regularly with key stakeholders involved in the implementation and delivery of the service to reflect on shared learning, agreed action and the impacts of change. This includes technology innovators, members of staff from the Test Bed hubs, voluntary sector providers and others. Numbers for these meetings are anticipated to be around 20 people, but should numbers increase beyond this we will ask stakeholders to nominate representatives to participate in the meetings and act as two-way conduits for the learning from their particular stakeholder group.

Action learning meetings will take place at three-monthly intervals and will involve a cycle of discussion, shared learning and reflection and agreed action to be taken forward regarding the operation, delivery and effectiveness of the service. Agreed action from these meetings will build on this cycle of learning and assessment of the service throughout the Test Bed period. To facilitate discussion at these meetings, key stakeholders are asked to complete weekly diaries documenting their experiences of the service, including what works well and less well, and any observations of patients' responses to the

For peer review only - http://bmjopen.bmj.com/site/about/guidelines.xhtml

service (time spent/support need/technical difficulties). Diaries are brief and simple to complete and are designed to feed into the action research meetings and to fine-tune themes for the focus groups and deliberative panels. The diaries are submitted electronically with participants being encouraged to keep a personal copy as an 'aide memoire' prior to each action learning meeting.

Toward the end of the evaluation we will also hold eight focus groups (N=6-10 participants per focus group) with key stakeholders involved in the delivery of the service. Our focus here is on recruiting not only those directly involved in delivering the service to older participants but also those organising and managing it. Recruitment to the focus groups will be split evenly across the two sites. The aim here will be to gain an in-depth understanding of how Fylde Coast Vanguard/Better Care Together services with the technology is impacting on working practices. The focus groups will also explore the extent to which the programme may help to increase: communication between the care teams; productivity; capacity and co-ordination of care; and overall work satisfaction.

For ease of reference, a summary of the overall evaluation methods is set out in Table 4 below.

Table 4: Summary of overall evaluation methods

Participants	Survey	Observational interview	Focus groups	Diaries	Action Learning meetings	Deliberative panels
Patients (with carers as appropriate)	Yes (3 over 6 months)	Yes (2 over 6 months)	X	X	X	A sample
Staff delivering service	X	X	Yes	Yes	Yes	A sample
Other key stakeholders (technology innovators, voluntary sector etc)	X	X	Yes	X	Yes	A sample
Managers/administrators	X	X	Yes	X	X	A sample

ETHICS AND DISSEMINATION

Audio recording

All qualitative data is being audio recorded using an encrypting digital voice recorder. In the process of transcription, names of participants are anonymized and any identifying features removed or coded. These points are included in the consent forms and are discussed with each participant before the interview/action research meeting/focus group/deliberative panel. All audio files will be deleted from the recorder once data analysis is complete.

Data analysis

The qualitative data will be analysed thematically with the aid of Atlas.ti qualitative software. Initial identification of emerging themes and initial coding will be undertaken by the field researcher then shared and agreed during evaluation team data analysis workshops. This will allow for the verification of the coding framework and enable the evaluation team to develop an analysis of the situation and understand the phenomenon being explored.[10] The major codes emerging from the data will be presented to the deliberative panels for fine-tuning and final verification.

Appendix 1 contains information regarding participant payment, data storage, participant benefit, potential risk to participants, withdrawal and researcher risk.

For peer review only - http://bmjopen.bmj.com/site/about/guidelines.xhtml

Deliberative panels

In the final three months of the evaluation, we will hold two deliberative panels (one per Vanguard site: N=15-20 per panel) with key stakeholders to include: older participants; members of the Vanguard care teams; key stakeholders from clinical and community settings, and social care; innovators; and senior managers and commissioners from the Test Bed sites.

Deliberative panels are designed to validate and fine-tune outcomes by producing an informed and collective view resulting from deliberation. They offer a bottom-up approach that can shift evaluation findings into outcomes related to policy, practice and guidance for employers. To achieve this, draft findings emerging from the data analyses will be presented with the aim of drawing on the various expertise of the participants.

In this case, it will help to identify and validate those key components of the intervention that will comprise a new model of care that may be scaled up for wider roll-out and will help to finalise the evaluation recommendations. The deliberative panels will also be used to begin preliminary discussions of how this model might form the basis of a joint health and social care strategy for telehealth/telecare. Finally, the panels will help us to fine-tune the Logic Model through a process of 'backward mapping' (see below).

The Logic Model framework

The evaluation is located within a Logic Model framework, enabling us to understand the impact of the intervention. It also enables us to consider the processes, management and participation that may have implications for the Test Bed's success. In developing the Logic Model, we are considering the following key elements:

- Activities – the focus of our model is the New Models of Care with Technology intervention. The programme of work is considered in its context, including the partners of the project and the policies and procedures that are in place.
- Inputs – including the financial, human, organisational, and material resources of the project. This includes the following stakeholders: older adults taking part in the project; Fylde Coast Vanguard and Better Care Together teams; clinicians; GPs; community nurses; other health professionals; and innovator, voluntary and community sector input.
- Outcomes – the desired and actual results of the project and a Logic Model that will support roll out and scalability.

The Logic Model has been designed as a processual model that will be populated using data gathered in the diaries and action research meetings. The elements identified above will be fine-tuned iteratively over the two years of the intervention and finalised through a process of 'backward mapping' during the final deliberative panels. In this way, the final Logic Model, designed to assist with wider roll-out of the intervention, will be developed by drawing on informed and collective views resulting from ongoing iteration and deliberation involving all key stakeholders.

Dissemination

The findings of the evaluation will be disseminated in the following ways:

For peer review only - http://bmjopen.bmj.com/site/about/guidelines.xhtml

a) A final evaluation report which also contributes to the national evaluation;
b) Presentations to all key stakeholders within the Vanguard sites and NHS England;
c) Conference papers and posters presented at regional, national, and international conferences;
d) Publication in a range of high quality, international, peer-reviewed scientific journals;
e) Publication in a range of journals/publications relevant to service providers;
f) Dissemination of evaluation highlights through social media.

FULL REFERENCES

1 George J & Martin F (2016) Briefing paper: Living with long-term conditions. British Medical Association. Available at: https://www.bma.org.uk/-/media/files/pdfs/.../living-with-long-term-conditions.pdf [Accessed on 6 April 2017].

2 King's Fund, The (2016) Quarterly Monitoring Report, No. 21. Available at: http://qmr.kingsfund.org.uk/2016/21?_ga=1.123564811.1874139399.1487344226/ (November 2016) [Accessed on 6 April 2017].

3 Local Government Association, The (2016) 'Adult Social Care Funding: 2016 State of the Nation Report'. November 2016. Available at: http://www.local.gov.uk/documents/10180/7632544/1+24+ASCF+state+of+the+nation+2016_WEB.pdf/e5943f2d-4dbd-41a8-b73e-da0c7209ec12 [Accessed on 6 April 2017].

4 NHS England (2017) 'About vanguards'. Available at: https://www.england.nhs.uk/ourwork/new-care-models/vanguards/about-vanguards/ [Accessed on 6 April 2017].

5 NHS Blackpool Clinical Commissioning Group (2017) 'Extensive Care'. Available at: http://blackpoolccg.nhs.uk/local-services/new-models-of-care/extensive-care/ [Accessed on 6 April 2017].

6 Bay Health and Care Partners (2017) 'Guide to Better Care Together'. Available at: http://clinicalimages.web.furness.net/files/BCT-Guide-13Jul.pdf [Accessed on 6 April 2017].

7 van Berkel C, Smith M, Horsfield D. et al. Evidence for supported self-care at scale: A population approach to evaluating technology enabled support for long term condition management. Liverpool, UK: Philips and Liverpool Clinical Commissioning Group 2016.

8 van Berkel C, Smith M, Horsfield D. et al. Evidence for supported self-care at scale. *International Journal of Integrated Care* 2016 16(5) DOI: dx.doi.org/10.5334/ijic..2595

9 Wennberg D, Siegel M, Darin B, et al. Combined Predictive Model: Final report and technical documentation. London, UK: The King's Fund 2006.

10 Bowling A. Research Methods in Health: Investigating Health and Health Services, Fourth Edition. Maidenhead, UK: Open University Press 2014.

For peer review only - http://bmjopen.bmj.com/site/about/guidelines.xhtml

AUTHORS' CONTRIBUTIONS

Sandra Varey is the principal author and wrote the majority of the manuscript. Alejandra Hernández, Thomas Palmer, Céu Mateus, Joann Wilkinson, Mandy Dixon and Christine Milligan are co-authors and each contributed to the editing and revision of the manuscript through several iterations. Christine Milligan is the Principal Investigator for the evaluation and is the corresponding author.

FUNDING STATEMENT

This work is supported by NHS England.

COMPETING INTERESTS STATEMENT

The authors declared no potential conflicts of interest with respect to the research, authorship, and/or publication of this article.

Acknowledgements

The authors wish to thank Amanda Thornton, the Patient by Experience groups, the Clinical Reference group and the LCIA Test Bed Evaluation Advisory Board for their helpful feedback on the initial design of the protocol materials.

In the original bid documentation, Philips contributed to the development of Table 2 presented in this paper.

For peer review only

Appendix 1

Participant payment

Participants taking part in the qualitative elements of the study will receive a voucher of £20 as a thank you in recognition of the time they have given to taking part in the evaluation. In the quantitative elements of the evaluation we are unable to offer any incentive because of the large numbers of participants.

Data storage

Personal data is being stored on a password safe university computer accessible only to the researchers. The research data is stored in an encrypted file using the software TrueCrypt.

All data is being stored securely in line with the Data Protection Act [11] and will be kept no longer than necessary.[12] Written data will be kept for a maximum of 10 years in a secure cabinet. Any electronic copies of data will be kept on the University server, which is a password protected system and will be encrypted. If electronic data requires transfer, this is done on an encrypted USB device. Any emails sent from participants (e.g. electronic diaries) are sent to a password secured email address that has been set up for the purpose of the study. Emails are saved electronically with any other electronic copies of data, with the email then being deleted. Where emails and interview/focus group data are printed for the purposes of analysis, they are stored with other written data in a secure cabinet. At the end of the study, the email account will be deleted. The paper copies of any participant contact details will be scanned and stored electronically, and paper copies destroyed.

Participant benefit

Participants have the direct benefit of using the technology during the two years of the Test Bed; however the Test Bed team recognise the need to make clear for those in Cohorts 1 and 4 that this is time-limited. Patients (particularly those in cohorts 2 and 3) for whom the intervention is a text messaging system or an app, will have access to these beyond the six month timeframe of the Test Bed programme. Given a key objective of the service is to enhance patient activation and ability to self-care, it is also anticipated that potential ongoing benefits will support older people with chronic health problems to remain in their own homes longer.

Patients and health professionals also have an opportunity to share directly with researchers their experiences of using and implementing this model of care. In this way, all participants can take an active part in shaping current and future healthcare services in place for older people with chronic conditions or dementia who are living at home.

Potential risk to participants

For patients participating in Phase 1 of the evaluation, there are no anticipated risks from participation in the project. All validated tools are validated for completion by proxy, and patients and family

For peer review only

members are therefore made aware that the patient can be supported to complete the surveys if required.

For patients taking part in Phase 2 of the evaluation research, no specific risks are anticipated. However, the following considerations have been made:

1. It is possible that a patient may become distressed by the presence of the researcher in their home during the observational interviews. To avoid this risk, the researcher will take care to check with the patient at the beginning of the interview to ensure they are happy to continue taking part in the study. The researcher will also do this at the end of the session and for the second interview.

2. If a patient should become distressed for any reason during an observational interview, the researcher will provide the patient with the contact details of different support networks for older people, as outlined in the participant information sheet. A member of the Test Bed hub will also be alerted should this be necessary, in order to assess whether a visit from a member of the hub is required.

Participants involved in the action learning meetings and the focus groups will be reminded that discussion in these settings is confidential and information shared (other than the shared and agreed action learning) should not be discussed with others outside of the meeting.

Withdrawal

Participants may choose to leave the study at any time and are assured that their data can be withdrawn up to four weeks after collection. The rationale for this four week time period is that, beyond four weeks, analysis will have begun. Those participating in focus groups are advised that, although every effort will be undertaken to remove their data should they request it during the timeframe, this may not always be possible. This is due to difficulties in identifying/attributing specific comments to individuals and because focus group data is often the outcome of group discussion, making it hard to distil to whom individual thoughts/comments should be attributed.

Researcher risk

There is limited risk to the quantitative researchers as they have very limited direct engagement with the participants (survey completion is largely through technology or telephone).

The qualitative researcher is interviewing participants in their own homes and is experienced in undertaking this type of fieldwork. The researcher is adhering to the University's lone worker policy. In order to minimise any potential risks, for each home visit conducted, the researcher identifies a 'buddy' (a member of staff within the University) with whom to remain in contact with: before the interview commences; when the interview has been terminated; and when the researcher has left the patient's home. The buddy is informed of the time and place of the interview, along with a contact telephone number.

Researchers within the team have the opportunity to debrief with other members of the team if required and have full access to counselling services if necessary.

References

For peer review only

11 Data Protection Act (1998) Data Protection Act. Available at: http://www.legislation.gov.uk/UKPGA/1998/29/contents [Accessed on 17 February 2017].

12 Bryman A. Social Research Methods, Sixth Edition. Oxford, UK: Oxford University Press 2016.

For peer review only

How effective and cost effective are innovative combinatorial technologies and practices for supporting older people with long-term conditions to remain well in the community? An evaluation protocol for an NHS Test Bed in Northwest England.

Journal:	*BMJ Open*
Manuscript ID	bmjopen-2017-017268.R2
Article Type:	Protocol
Date Submitted by the Author:	28-Nov-2017
Complete List of Authors:	Varey, Sandra; Lancaster University, Division of Health Research<br>Hernândez, Alejandra; Lancaster University<br>Palmer, Tom; Lancaster University, Department of Mathematics and Statistics ; University of Warwick, Warwick Medical School, Division of Health Sciences<br>Mateus, Ceu; University of Lancaster, Division of Health Research<br>Wilkinson, Joann; Lancaster University<br>Dixon, Mandy; Lancaster University<br>Milligan, Christine; Lancaster University
**Primary Subject Heading**:	Health services research
Secondary Subject Heading:	Geriatric medicine
Keywords:	Health technologies, older people, long-term conditions, patient activation, self-care

For peer review only

Title: How effective and cost effective are innovative combinatorial technologies and practices for supporting older people with long-term conditions to remain well in the community? An evaluation protocol for an NHS Test Bed in Northwest England.

Sandra Varey[1], Alejandra Hernández[1], Tom Palmer[2], Céu Mateus[3], Joann Wilkinson[1], Mandy Dixon[4] and Christine Milligan[1]

1. Centre for Ageing Research, Division of Health Research, Lancaster University, Lancaster LA1 4YG, United Kingdom

2. Department of Mathematics and Statistics, Lancaster University, Lancaster LA1 4YG, United Kingdom

3. Health Economics, Division of Health Research, Lancaster University, Lancaster LA1 4YG, United Kingdom

4. Lancaster Health Hub, Lancaster University, Lancaster LA1 4YG, United Kingdom

Correspondence to:

Professor Christine Milligan

Centre for Ageing Research

Division of Health Research

Lancaster University

Lancaster LA1 4YG

United Kingdom

Email: c.milligan@lancaster.ac.uk

Telephone: +44 1524 592128

Word count: 4551

For peer review only

Abstract

Introduction:
The Lancashire and Cumbria Innovation Alliance (LCIA) Test Bed is a partnership between National Health Services in England (NHS), industry (led by Philips) and Lancaster University. Through the implementation of a combination of innovative health technologies and practices, it aims to determine the most effective and cost effective ways of supporting frail older people with long-term conditions to remain well in the community. Among the Test Bed's objectives are to: improve patient activation and the ability of older people to self-care at home; reduce healthcare system utilisation; and deliver increased workforce productivity.

Methods and Analysis:
Patients aged 55 years and over are recruited to four cohorts defined by their risk of hospital admission, with long-term conditions including COPD, dementia, diabetes and heart failure. The programme is determined on an individual basis, with a range of technologies available. The evaluation is adopting a two-phase approach: Phase 1 includes a bespoke patient survey and a mass matched control analysis; and Phase 2 is using observational interviews with patients, and weekly diaries, action learning meetings and focus groups with members of staff and other key stakeholders. Phase 1 data analysis consists of a statistical evaluation of the effectiveness of the programme. A health economic analysis of its costs and associated cost changes will be undertaken. Phase 2 data will be analysed thematically with the aid of Atlas.ti qualitative software. The evaluation is located within a logic model framework, to consider the processes, management and participation that may have implications for the Test Bed's success.

Ethics and Dissemination:
The LCIA Test Bed evaluation has received ethical approval from the Health Research Authority and Lancaster University's Faculty of Health and Medicine Research Ethics Committee. A range of dissemination methods are adopted including deliberative panels to validate findings and develop outcomes for policy and practice.

Word count: 300

For peer review only

For peer review only - http://bmjopen.bmj.com/site/about/guidelines.xhtml

Strengths and limitations of this study

- The evaluation is considering both the effectiveness, in terms of measures such as hospital admissions, and cost effectiveness of the Test Bed
- The protocol evaluates the potential for patient activation through health technology
- Observational interviews with patients in their own homes enable insights into how older people experience and engage with the technology
- For both the Test Bed participants and controls the evaluation has to use proxy measures of the costs incurred to the NHS
- Due to the timescale of the overall programme, it is not possible to assess the longer-term impact of the programme

Key words

Health technologies; older people; long-term conditions; patient activation; self-care.

For peer review only

INTRODUCTION

In the UK, increasing numbers of older people are living with long-term conditions including diabetes, chronic obstructive pulmonary disease (COPD), heart failure and dementia.[1] This is contributing to increased pressure on health and social care systems including accident and emergency services, which in turn increases demand on the number of hospital beds.[2] At the same time, local authorities are experiencing a reduction in the funding available to provide social care.[3]

Concerns about this so-called care-gap and our future ability to cope with the growing numbers of older people with long-term conditions underpins governmental drivers to develop new models of care within Vanguard sites[1] that are cost-effective and which facilitate older people's ability to better self-manage their care needs at home. The NHS England Test Bed programme has thus been designed to encourage the trialling of new models of care that are supported by health technologies across a number of areas in the UK.

This paper reports on the protocol for the evaluation of one such programme, the Lancashire and Cumbria Innovation Alliance (LCIA) Test Bed in the north-west of England. The programme commenced on 1st April 2016 and is due to be completed on 30th June 2018.

The LCIA Test Bed initiative

The LCIA Test Bed[2] is a partnership between the NHS, industry (led by Philips) and Lancaster University, and is one of seven Test Beds located across England. Two neighbouring Vanguard sites, part of the Lancaster Health Hub (an established NHS/University partnership comprising ten local organisations), will deliver the Test Bed. The Test Bed's aims and objectives are presented below.

Test Bed Aims:
To determine the most effective and cost effective ways of supporting frail older people with dementia and other long-term conditions (LTCs) to remain well in the community and avoid unnecessary hospital admissions.

Test Bed Objectives:
To use a combinatorial range of technologies and services to:

- better support frail older people, older people living with long-term conditions;
- improve patient activation[3] and the ability of older people with a range of LTCs to self-care at home;
- improve health awareness and outcomes for older people with LTCs;
- reduce healthcare system utilisation and increase productivity within the healthcare workforce.

---

[1] In 2015, the National Health Service in England selected 50 vanguard sites to take a lead on the development of new integrated care models designed to act as blueprints for the future of the NHS. Complete redesign of whole health and care systems are being considered. The hope is that the vanguards will provide inspiration to the rest of the health and care system across the UK (NHS England, 2017).[4] For more information about NHS Vanguards, visit: https://www.england.nhs.uk/ourwork/new-care-models/vanguards/

[2] For more information about the LCIA Test Bed, visit: https://www.england.nhs.uk/ourwork/innovation/test-beds/innovation-alliance/

[3] For more information about the NHS definition of patient activation, visit: https://www.england.nhs.uk/ourwork/patient-participation/self-care/patient-activation/pa-faqs/

For peer review only - http://bmjopen.bmj.com/site/about/guidelines.xhtml

Over two years, the Test Bed is implementing a combination of innovative technologies and practices aimed at supporting these aims and objectives. The clinical challenge is to reduce hospital admissions, create capacity, reduce overall costs, minimize other health and social care usage, and maximize patient outcomes for a targeted population of older people (defined in this Test Bed as those aged 55 years and over) who suffer a range of long term conditions including dementia. These LTCs present a major challenge for the area given its dispersed population, and innovative solutions are urgently required.

The main technology partner is Philips who, together with a number of small and medium sized enterprises (SMEs), social enterprises, and voluntary organisations, is working with the Test Bed to introduce a health technology-enabled supported self-care programme.[4]

The two Vanguard sites include the Fylde Coast and North Lancashire, with the technologies being provided in addition to the new models of care being implemented by these two sites. The Fylde Coast Vanguard[5] is drawing on the Extensive Care model, which brings together a range of services in the same location to achieve a co-ordinated team of healthcare professionals, with patients each allocated a wellbeing support worker.[5] This model focuses on multispecialty community providers, moving specialist care out of hospitals and into the community.[6] The North Lancashire Vanguard, Better Care Together, sees healthcare professionals working together in partnership to help people manage their own health conditions.[7] This model focuses on integrated primary and acute care settings, joining up GP, hospital, community and mental health services.[8] Further details of these new models of care are set out in the NHS England 'Five Year Forward View' [9] and by the Nuffield Trust [10].

As part of the LCIA Test Bed programme, these existing services are being combined with innovative technologies and support for older people with LTCs including COPD, Heart Failure, Diabetes and Dementia. For those with lower levels of risk in cohort 3 (see Table 1), asthma and hypertension will also be included. The focus of this Test Bed is to empower patients to actively manage their health conditions and change behaviour through the provision of appropriate technologies, coaching and education.

A core part of the programme also focuses on increasing involvement of the third sector and community organisations in providing support for the Test Bed model, and understanding the impact of the service on staff and key stakeholders.

For peer review only

Core outcomes for the evaluation

The Test Bed focuses on a combinational approach to technologies. Hence, rather than focusing on the impact of any single technology, the Test Bed evaluation is focusing on how health technologies as an addition to existing Vanguard services can improve and promote self-care at home and reduce hospital admissions for older people with LTCs.

The evaluation is focusing on three primary outcomes:

1. The extent to which supported self-care telehealth technology can improve patient outcomes, through reduced hospital admissions, medication, GP visits, home visits and community

[4] Cambridge Cognition Limited; Good Things Foundation; Intelesant Limited; MKS Solutions Limited; Simple Shared Healthcare Limited; and uMotif Limited.

[5] The Fylde Coast Vanguard encompasses NHS Blackpool CCG, NHS Fylde & Wyre CCG and Blackpool Teaching Hospitals.

For peer review only - http://bmjopen.bmj.com/site/about/guidelines.xhtml

service use;

2. The extent to which supported self-care telehealth technology can improve patient activation for frail older people with LTCs;

3. The cost effectiveness of the programme and how it might be scaled up to provide better value for patients and taxpayers.

Secondary outcomes include:

1. Identification of the strengths and weaknesses of the programme and how these may be built on or addressed;

2. Assessment of patient/staff perceptions of how the new service improves on existing services and makes best use of voluntary and community services;

3. Assessment of the impact of the new service within the healthcare workforce in terms of communication between care teams, productivity, capacity, co-ordination of care, and work satisfaction.

To achieve this, we are:

a) Using quantitative and qualitative measures to examine benefits to patients and patient outcomes as identified above;

b) Undertaking an economic evaluation of the impact of adding a combination of technologies to support and promote self-care in the provision of healthcare in comparison to standard care with Cohorts 1 and 2 (see Table 2 below);

c) Drawing on a Logic Model to demonstrate how to construct the service as a scalable model that might provide better value to patients and taxpayers.

METHODS AND ANALYSIS

Ethical approvals

The overall evaluation involves two distinct phases: Phase 1 involves collection of survey data designed to evaluate any change in health and health-related behaviours in patients using the combinatorial technologies; and Phase 2 involves understanding the impact and experiences of patients using the technologies, and the impact on working practices amongst members of staff involved in the delivery of the technologies. Following discussion with the Health Research Authority (HRA) and the Research and Development Directors of the NHS Trusts involved in the programme, it was agreed that Phase 1 was Service Evaluation and as such did not require HRA approvals. Phase 2 however was deemed by the HRA to be gathering new data and was thus defined by them as ‘research’, therefore requiring HRA approvals (IRAS Project ID: 208395). We received ethics approval from Lancaster University’s Faculty of Health and Medicine Research Ethics Committee for Phase 1 (Reference: FHMREC16025). Further details about the two phases are provided below.

For peer review only - http://bmjopen.bmj.com/site/about/guidelines.xhtml

Phase 2 of the evaluation has also been adopted onto the National Institute for Health Research portfolio.[6]

Sampling and recruitment

Patients are being recruited to four cohorts defined by their risk of hospital admission. The long-term conditions are noted above and are outlined in Table 1.

Table 1: Recruitment criteria for the four LCIA Test Bed cohorts

Cohort	Age	Risk of hospital admission	Long-term conditions
Cohort 1	Aged 55 years or over	>25%	COPD, HF
Cohort 2		>10% and <25%	
Cohort 3		<10%	Diabetes, Asthma, CHD, Hypertension
Cohort 4		N/A	Early stage dementia (ACE-III assessment tool)

Participants for cohorts 1, 2 and 3 are being recruited to the Test Bed through the Vanguard services to which they are already assigned. As illustrated in Table 1, Cohort 4 is focusing specifically on patients with early stage dementia. Potential participants for Cohort 4 are being recruited through the Memory Assessment Service (MAS) in the Fylde Coast and North Lancashire. In the diagnosis of dementia, these services use the Addenbrooke's Cognitive Examination-III (ACE-III) assessment tool to focus on five cognitive domains. People who have received a recent diagnosis of mild dementia by a consultant psychiatrist following their comprehensive assessment with the MAS are deemed as having fulfilled the entry criteria of mild dementia. For these patients, the ACE-III or Mini-ACE has been completed as part of their cognitive assessment at the MAS clinic, the results of which have aided the formulation of the dementia diagnosis. Only patients judged by a clinician to have mild dementia are invited to take part in the Test Bed.

All patients consenting to the take part in the Test Bed and receiving the technology are required to participate in Phase 1 of the service evaluation and will sign a consent form. Participation in Phase 2, the qualitative phase of the evaluation is optional. Patients indicating an interest in participating in Phase 2 are being consented separately through a two-stage consent process.

The sample size for recruitment to each cohort has been informed by population and risk data for the area, along with the methodology used in a previous study.[11,12] As Table 2 illustrates, the overall aim of the Test Bed is to recruit 1600 patients; however, given the anticipated challenges of recruitment and potential attrition due to the nature of the patient group, we estimate an overall recruitment and retention figure of 60% (overall N=960).

Table 2: LCIA Test Bed cohort sample sizes (*combined over a population of app. 310,000 people)[7]

[6] NIHR Portfolio reference number is the same as the IRAS number (IRAS Project ID 208395)

[7] See Acknowledgements

For peer review only - http://bmjopen.bmj.com/site/about/guidelines.xhtml

Cohort	Percent of the total population*	Total patients	Percent of frail and elderly patients	Percent of patients with dementia	Number of patients estimated to be appropriate for the service	Percent of appropriate patients within cohort that will be recruited within the Test Bed period	Minimum number of patients recruited within Test Bed period	Estimated number of patients by the end of the Test Bed period
**1** (age>55, risk>25% & 1 LTC)	1.70%	5,262	20%	3.5%	1,737	30%	521	500
**2** (age>55, 10%>risk<25 % & 1 LTC)	3.00%	9,286	4%	1.1%	3,064	15%	460	500
**3** (age>55, 10%<risk 1 of a broader set of LTCs)	9.20%	28,477	2%	1.6%	9,398	5%	470	500
**4** (diagnosis of dementia)	0.79%	2,430		100.0%	810	10%	81	100

Allowing for a phased increase in recruitment, the overall recruitment is estimated at approximately 60-70% of the figures identified in Table 2. This will provide sufficient data from cohorts 1 and 2 to demonstrate a change in hospital admissions (a primary outcome). Cohorts 3 and 4 will not be assessed for this outcome, although other measures of wellbeing, patient activation, and loneliness will be used to describe these cohorts. The secondary outcomes are being supported by observational interviews across all four cohorts.

The programme

The patient interface varies across the four cohorts, and the technologies available to people within the different cohorts are presented in Table 3. A patient's programme is determined on an individual basis and is informed by a number of factors, including health condition(s), personal preferences and other needs. Some patients are using different technologies at different periods of time within the six month programme.

Table 3: Technologies available to each cohort

Cohort	Company	Technology	Description
1 & 4	Philips	Motiva[8]	A telehealth platform which operates through a tablet or TV set top box interface that connects wirelessly to a range of tele-monitoring equipment in the home (e.g. wireless weighing scales, blood pressure meters, pulse oximeters and thermometers).
	MKS Solutions Limited	Speakset[9]	A video calling system that connects a health professional with a patient through the patient's TV in their own home.

[8] http://www.philips.co.uk/healthcare/solutions/enterprise-telehealth/home-telehealth
[9] http://www.speakset.com

For peer review only - http://bmjopen.bmj.com/site/about/guidelines.xhtml

For peer review only

	NHS Stoke-on-Trent Clinical Commissioning Group's Licence	Florence/NHS Simple[10]	A short message communication software providing a reminder/communication text messaging service for patients.
1, 2 & 3	Cambridge Cognition Limited	CANTAB Mobile[11]	An assessment tool designed for healthcare professionals to identify the earliest signs of clinically significant memory impairment. The assessment comprises three tests: the Paired Associates Learning (PAL) test to assess episodic memory; the Geriatric Depression Scale (GDS) to identify signs of depression and an Activities of Daily Living questionnaire (ADL) to assess functionality in daily life.
2	Intelesant Limited	Intelesant[12]	A mobile app to support daily management of COPD through SMS text messaging alerts for coaching, prompts, and reminders. It incorporates the individual's action plan in response to changes in symptoms.
	Philips	Personal Blood Pressure Cuff[13]	A blood pressure monitor that works in conjunction with the Philips Suite App to enable a patient to record their blood pressure.
2 & 4	Philips	Health Watch[14]	A health watch that tracks heart rate and other cardio condition metrics, monitors activity, sleeping patterns, and nutrition behaviour (not a sports watch). It is best paired with a smartphone running the Philips Suite App.
3	uMotif Limited	uMotif[15]	A health app available as a mobile app, digital tool or wearable device to capture data through a graphical interface and help patients track and understand their health and symptoms. It provides a health report, connects to other wearable devices, sends medication reminders, and keeps track of regular tasks and daily activities.
4	National Museums Liverpool	House of Memories[16]	A health app that enables people living with dementia and their carers/families to keep a record of objects and experiences from the past.<br>Patients and/or carers can create their own memory tree, memory box or memory timeline.

In summary, the LCIA Test Bed provides a structured programme of case management, monitoring, education and coaching supported by a clinical hub. It thus involves clinical and non-clinical teams, community involvement and community feedback. Good Things Foundation[17] is providing support to patients regarding their digital skills to help them use the innovator technologies.

[10] http://www.simple.uk.net
[11] http://www.cambridgecognition.com
[12] http://www.intelesant.com
[13] http://www.philips.co.uk/c-m-hs/health-programs/upper-arm-blood-pressure-monitor
[14] http://www.philips.co.uk/c-m-hs/health-programs/health-watch
[15] http://www.umotif.com
[16] http://www.liverpoolmuseums.org.uk/learning/projects/house-of-memories/
[17] http://www.goodthingsfoundation.org

For peer review only - http://bmjopen.bmj.com/site/about/guidelines.xhtml

Evaluation design

A requirement of this evaluation[18] was that it adopt a rapid cycle approach, to enable the delivery of the programme to be adapted and amended in the light of feedback received from patients and clinicians. As a result, a randomised controlled trial design was deemed unsuitable.

To address the core aims and objectives outlined above and the NHS England requirements, the evaluation was designed as a longitudinal, mixed method evaluation, to include:

Phase 1:
1. A bespoke patient survey incorporating validated tools;
2. A mass matched control analysis.

Phase 2:

3. Phased observational interviews with patients;
4. Weekly diaries, action learning meetings and focus groups with staff and other key stakeholders.

The two phases of the evaluation design are adopting different sampling and recruitment strategies, along with methods, as set out below. Each has its own participant information sheets, consent forms and, where applicable, schedules (e.g. for the interviews and focus groups). A separate flyer has been designed for patients with dementia, providing information about the study in an informative and accessible manner, drawing on images and large font. In all instances, it will be made clear to patients that they can be supported by a carer or other family member should they wish. It is anticipated that all participants in cohort 4 (mild dementia) will be accompanied by a carer or other family member.

All materials developed for the evaluation and submitted to ethical review were shared and informed through discussion with: patient by experience groups (including those with dementia); a clinical reference group; and an evaluation advisory board. Feedback and comment from these three groups was taken into account in finalising the documents submitted for ethics approval.

1. The patient survey

Recruitment and informed consent

In line with referral to the service, recruitment is being staggered over the programme period. Only patients judged by a clinician to have an acceptable level of cognitive function to give informed consent are invited to take part in the Test Bed. The impact of the programme on each participant is being evaluated over a six month period. Final recruitment to the evaluation will thus be at the end of month 15, to allow for the six month programme and a three-month period for final analyses. All patients recruited into the Test Bed and receiving the service are included in the quantitative (service evaluation) stage, and are completing the Phase 1 consent form. Patients in Phase 1 are completing a baseline, mid (12 weeks) and endpoint (24 weeks) survey during the six month programme.

Methods

The data analysis consists of a statistical evaluation of the effectiveness of the programme and a health economic analysis of its costs and associated cost changes for Cohorts 1 and 2. The evaluation is

[18] Stipulated by NHS England

For peer review only - http://bmjopen.bmj.com/site/about/guidelines.xhtml

collecting anonymised information for each participant concerning: age, gender, ethnicity, living arrangements, education completed, and use of health care services in the four weeks before completing the questionnaire such as hospital services (inpatient care, outpatient visits, and A&E visits), primary care services, social care services, and medicines. In all cohorts, validated instruments for use with older people to assess health related quality of life, health and wellbeing and patient activation are being used (each of can be completed by proxy if required): Warwick-Edinburgh Mental Wellbeing Scale (WEMWBS), EQ-5D-5L, Patient Activation Measure (PAM)13, and the De Jong Gierveld Loneliness Scale. These tools are reapplied at weeks 12 and 24.

The evaluation team are working with the technology innovators[19] to minimise the demands of the evaluation on patients. Participants are also being offered the option of completing the questionnaires on paper or by telephone.

Drawing on HES data, the primary outcomes for cohorts 1 and 2 will be evaluated against a matched control group (3 controls to 1 Test Bed patient). This will be coordinated in conjunction with the Midlands and Lancashire Commissioning Support Unit.

For Cohorts 1 and 2, the quantitative analysis will consider a cost-effectiveness analysis of the programme through the estimation of the mean of hospital admissions for the Test Bed patients and for the control group. The primary outcome is the difference between the change in the two groups which will be estimated using linear regression adjusted for the matching covariates. Ninety-five percent confidence intervals will be reported for all estimated coefficients. ;

2. The mass control group

The evaluation team will match 3 control subjects per programme participant for Cohorts 1 and 2, the controls being sampled from the same Vanguard Sites as those on the Test Bed. In terms of contamination between control and Test Bed groups, obtaining control data from the Commissioning Support Unit ensures no controls are also members of the Test Bed. The matching will be performed using the following variables: predicted probability (risk) of hospital admission, long-term condition, sex, and age. The predicted probability of hospital admission for each participant and control will be calculated using the Combined Predictive Model.[13] The matching will be performed using a standard multivariate matching algorithm (Mahalanobis distance matching). The aim of matching on these variables is to obtain a control group which has similar characteristics for these variables compared to the programme participants. This should help minimise any possible confounding effects of these variables.

The statistical analysis is conducted using the statistical software Stata (version 14).

3. Phased observational interviews with patients

Recruitment and informed consent
Patient participation in Phase 2 of the evaluation is optional and participants are asked to indicate their preference for this in the Phase 1 consent form. Based on past experience, it is anticipated that more patients will indicate a willingness to take part in the second phase than will be required. Where possible, we are seeking to purposively sample on the basis of: chronic health condition(s) (cohorts 1-

[19] The term 'innovators' has been coined by NHS England for the purposes of the Test Beds and refers to technology companies deemed to be working at the cutting edge of digital health design and development.

For peer review only - http://bmjopen.bmj.com/site/about/guidelines.xhtml

3); age (55-64; 65-74; 75-84; ≥85); gender; ethnicity; postal address (based on the ONS deprivation scale); and co- versus lone dwelling. Ten to fifteen patients are being recruited from each cohort to participate in the in-depth qualitative phase of the evaluation (N = 40-60).

Those participating in Phase 2 are being consented at the time of their first interview. A separate flyer has been designed for patients with dementia, to provide information about the Test Bed in an informative and accessible manner. To ensure informed consent and that each participant understands the consent form, the researcher discusses each aspect of the consent form with each participant before it is signed.

Ethical approval was received from the HRA for an alternative consent form with a large font size, and for which clauses on the consent from can be cut into strips, with one clause per strip of paper. This enables the researcher to go through each clause with the participant, focusing on one strip of paper at a time. Once the participant understands the clause clearly, they are asked to initial the clause discussed. This option makes the information in the consent form more manageable for the patient and, prior to ethical approval, was discussed and approved by the Lancaster patient experience group (linked to the NHS). When consenting a person to Cohort 4, the researcher talks through the consent form with the patient and in the presence of a family member, friend or carer.

Methods

Two observational interviews are being undertaken with each participant in their own home over the six month period of the programme: a) in the first month of participating in the programme; and b) during the final month 6. The home-base setting is important as it facilitates a better understanding of participants' health status, health knowledge and activation prior to their participation in the programme, how they engage with the service and use the technology within their own homes at the outset and whether this changes over time. This approach is specifically designed to gain a better understanding of: how participants engage with and experience the technology and Fylde Coast Vanguard / Better Care Together services; how this may influence patient activation and self-management of care; any barriers to improving self-care; whether the programme can increase their sense of empowerment and independence; service satisfaction; and overall quality of life.

4. Understanding the impact on staff and key stakeholders: diaries, action learning meetings and focus groups

In the early stage of the Test Bed, interviews and focus groups will be conducted with key stakeholders to determine the lessons learned in the process of operationalising the Test Bed. This data will begin to inform and help map out the logic model (see below). Informed consent will be taken for all participants.

All key stakeholders and service providers involved in the delivery of the service are also invited to complete brief weekly diaries and participate in regular 'action learning' meetings, informed by the diary data, as part of a rapid cycle review process. Here, members of the evaluation team meet regularly with key stakeholders involved in the implementation and delivery of the service to reflect on shared learning, agreed action and the impacts of change. This includes technology innovators, members of staff from the Test Bed hubs, voluntary sector providers and others. Numbers for these meetings are anticipated to be around 20 people, but should numbers increase beyond this we will ask stakeholders to nominate representatives to participate in the meetings and act as two-way conduits for the learning from their particular stakeholder group.

Action learning meetings will take place at three-monthly intervals and will involve a cycle of discussion, shared learning and reflection and agreed action to be taken forward regarding the operation, delivery

For peer review only - http://bmjopen.bmj.com/site/about/guidelines.xhtml

and effectiveness of the service. Agreed action from these meetings will build on this cycle of learning and assessment of the service throughout the Test Bed period. To facilitate discussion at these meetings, key stakeholders are asked to complete weekly diaries documenting their experiences of the service, including what works well and less well, and any observations of patients' responses to the service (time spent/support need/technical difficulties). Diaries are brief and simple to complete and are designed to feed into the action research meetings and to fine-tune themes for the focus groups and deliberative panels. The diaries are submitted electronically with participants being encouraged to keep a personal copy as an 'aide memoire' prior to each action learning meeting.

Toward the end of the evaluation we will also hold four focus groups (N=6-10 participants per focus group) with key stakeholders involved in the delivery of the service. Our objective here is to not only recruit those directly involved in delivering the service to older participants but also those organising and managing it. Recruitment to the focus groups will be split evenly across the two sites. The aim here will be to gain an in-depth understanding of how Fylde Coast Vanguard/Better Care Together services with the technology is impacting on working practices. The focus groups will also explore the extent to which the programme may help to increase: communication between the care teams; productivity; capacity and co-ordination of care; and overall work satisfaction.

For ease of reference, a summary of the overall evaluation methods is set out in Table 4 below.

Table 4: Summary of evaluation methods being applied to Test Bed participants

Participants	Survey	Observational interview	Focus groups	Diaries	Action Learning meetings	Deliberative panels
Patients (with carers as appropriate)	Yes (3 over 6 months)	Yes (2 over 6 months)	N/A	N/A	N/A	Sample
Staff delivering service	N/A	N/A	Yes	Yes	Yes	Sample
Other key stakeholders (technology innovators, voluntary sector, etc.)	N/A	N/A	Yes	N/A	Yes	Sample
Managers/administrators	N/A	N/A	Yes	N/A	N/A	Sample

ETHICS AND DISSEMINATION

Audio recording

All qualitative data is being audio recorded using an encrypting digital voice recorder. In the process of transcription, names of participants are anonymized and any identifying features removed or coded. These points are included in the consent forms and are discussed with each participant before the interview/action research meeting/focus group/deliberative panel. All audio files will be deleted from the recorder once data analysis is complete.

Data analysis

The qualitative data will be analysed thematically with the aid of Atlas.ti qualitative software. Initial identification of emerging themes and initial coding will be undertaken by the field researcher then shared and agreed during evaluation team data analysis workshops. This will allow for the verification of the coding framework and enable the evaluation team to develop an analysis of the situation and understand the phenomenon being explored.[14] The major codes emerging from the data will be presented to the deliberative panels for fine-tuning and final verification.

For peer review only - http://bmjopen.bmj.com/site/about/guidelines.xhtml

Appendix 1 contains information regarding participant payment, data storage, participant benefit, potential risk to participants, withdrawal and researcher risk.

Deliberative panels

In the final three months of the evaluation, we will hold two deliberative panels (one per Vanguard site: N=15-20 per panel) with key stakeholders to include: older participants; members of the Vanguard care teams; key stakeholders from clinical and community settings, and social care; innovators; and senior managers and commissioners from the Test Bed sites.

Deliberative panels are designed to validate and fine-tune outcomes by producing an informed and collective view resulting from deliberation. They offer a bottom-up approach that can shift evaluation findings into outcomes related to policy, practice and guidance for employers. To achieve this, draft findings emerging from the data analyses will be presented with the aim of drawing on the various expertise of the participants.

In this case, it will help to identify and validate those key components of the programme that will comprise a new model of care that may be scaled up for wider roll-out and will help to finalise the evaluation recommendations. The deliberative panels will also be used to begin preliminary discussions of how this model might form the basis of a joint health and social care strategy for telehealth/telecare. Finally, the panels will help us to fine-tune the Logic Model through a process of 'backward mapping' (see below).

The Logic Model framework

The evaluation is located within a Logic Model framework, enabling us to understand the impact of the programme. It also enables us to consider the processes, management and participation that may have implications for the Test Bed's success. In developing the Logic Model, we are considering the following key elements:

- Activities – the focus of our model is the New Models of Care with Technology programme. The programme of work is considered in its context, including the partners of the project and the policies and procedures that are in place.
- Inputs – including the financial, human, organisational, and material resources of the project. This includes the following stakeholders: older adults taking part in the project; Fylde Coast Vanguard and Better Care Together teams; clinicians; GPs; community nurses; other health professionals; and innovator, voluntary and community sector input.
- Outcomes – the desired and actual results of the project and a Logic Model that will support roll out and scalability.

The Logic Model has been designed as a processual model that will be populated using data gathered in the diaries and action research meetings. The elements identified above will be fine-tuned iteratively over the two years of the programme and finalised through a process of 'backward mapping' during the final deliberative panels. In this way, the final Logic Model, designed to assist with wider roll-out of the programme, will be developed by drawing on informed and collective views resulting from ongoing iteration and deliberation involving all key stakeholders. Figure 1 provides an overview of the Logic Model being used for the Test Bed evaluation.

For peer review only - http://bmjopen.bmj.com/site/about/guidelines.xhtml

Dissemination

The findings of the evaluation will be disseminated in the following ways:

a) A final evaluation report which also contributes to the national evaluation;
b) Presentations to all key stakeholders within the Vanguard sites and NHS England;
c) Conference papers and posters presented at regional, national, and international conferences;
d) Publication in a range of high quality, international, peer-reviewed scientific journals;
e) Publication in a range of journals/publications relevant to service providers;
f) Dissemination of evaluation highlights through social media.

FULL REFERENCES

1 George J & Martin F (2016) Briefing paper: Living with long-term conditions. British Medical Association. Available at: https://www.bma.org.uk/-/media/files/pdfs/.../living-with-long-term-conditions.pdf [Accessed on 20 November 2017].

2 King's Fund, The (2016) Quarterly Monitoring Report, No. 21. Available at: http://qmr.kingsfund.org.uk/2016/21?_ga=1.123564811.1874139399.1487344226/ (November 2016) [Accessed on 20 November 2017].

3 Local Government Association, The (2016) 'Adult Social Care Funding: 2016 State of the Nation Report'. November 2016. Available at: http://www.local.gov.uk/documents/10180/7632544/1+24+ASCF+state+of+the+nation+2016_WEB.pdf/e5943f2d-4dbd-41a8-b73e-da0c7209ec12 [Accessed on 20 November 2017].

4 NHS England (2017) 'About vanguards'. Available at: https://www.england.nhs.uk/ourwork/new-care-models/vanguards/about-vanguards/ [Accessed on 20 November 2017].

5 NHS Blackpool Clinical Commissioning Group (2017) 'Extensive Care'. Available at: http://blackpoolccg.nhs.uk/local-services/new-models-of-care/extensive-care/ [Accessed on 20 November 2017].

6 NHS England (2017) 'Multispecialty Community Provider Vanguards'. Available at: https://www.england.nhs.uk/ourwork/new-care-models/vanguards/care-models/community-sites/ [Accessed on 20 November 2017].

7 Bay Health and Care Partners (2017) 'Guide to Better Care Together'. Available at: http://clinicalimages.web.furness.net/files/BCT-Guide-13Jul.pdf [Accessed on 20 November 2017].

8 NHS England (2017) 'Integrated Primary and Acute Care Systems Vanguards'. Available at: https://www.england.nhs.uk/ourwork/new-care-models/vanguards/care-models/primary-acute-sites/ [Accessed on 20 November 2017].

9 NHS England (2017) 'Five Year Forward View'. Available at: https://www.england.nhs.uk/wp-content/uploads/2014/10/5yfv-web.pdf [Accessed on 20 November 2017].

For peer review only - http://bmjopen.bmj.com/site/about/guidelines.xhtml

10 Nuffield Trust (2017) 'New Models of Health Care Delivery'. Available at: https://www.nuffieldtrust.org.uk/our-priorities/new-models-of-health-care-delivery [Accessed on 20 November 2017].

11 van Berkel C, Smith M, Horsfield D. et al. Evidence for supported self-care at scale: A population approach to evaluating technology enabled support for long term condition management. Liverpool, UK: Philips and Liverpool Clinical Commissioning Group 2016.

12 van Berkel C, Smith M, Horsfield D. et al. Evidence for supported self-care at scale. *International Journal of Integrated Care* 2016 16(5) DOI: dx.doi.org/10.5334/ijic..2595

13 Wennberg D, Siegel M, Darin B, et al. Combined Predictive Model: Final report and technical documentation. London, UK: The King's Fund 2006.

14 Bowling A. Research Methods in Health: Investigating Health and Health Services, Fourth Edition. Maidenhead, UK: Open University Press 2014.

AUTHORS' CONTRIBUTIONS

Sandra Varey is the principal author and wrote the majority of the manuscript. Alejandra Hernández, Tom Palmer, Céu Mateus, Joann Wilkinson, Mandy Dixon and Christine Milligan are co-authors and each contributed to the editing and revision of the manuscript through several iterations. Christine Milligan is the Principal Investigator for the evaluation and is the corresponding author.

FUNDING STATEMENT

This work is supported by NHS England.

COMPETING INTERESTS STATEMENT

The authors declared no potential conflicts of interest with respect to the research, authorship, and/or publication of this article.

Acknowledgements

The authors wish to thank Amanda Thornton, the Patient by Experience groups, the Clinical Reference group and the LCIA Test Bed Evaluation Advisory Board for their helpful feedback on the initial design of the protocol materials.

In the original bid documentation, Philips contributed to the development of Table 2 presented in this paper.

Figure legends

Figure 1: Logic Model for Test Bed Evaluation

For peer review only - http://bmjopen.bmj.com/site/about/guidelines.xhtml

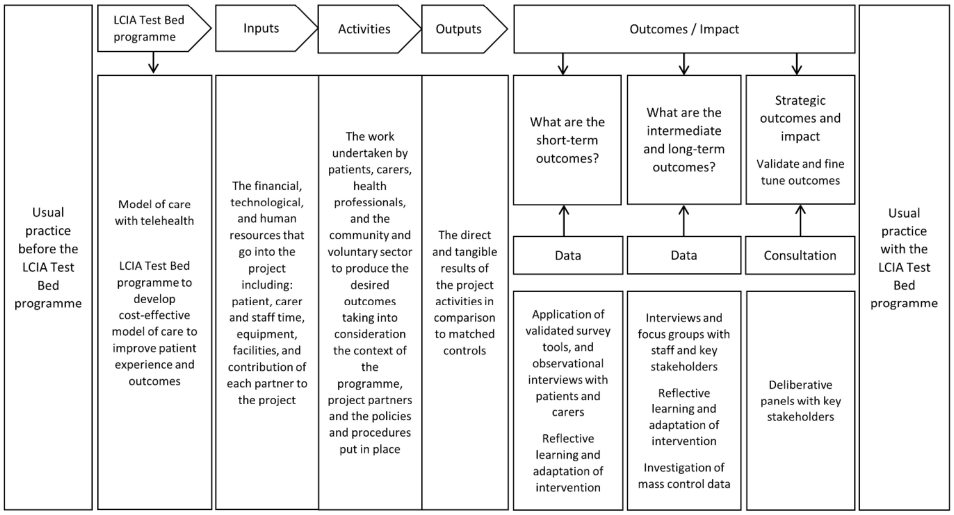

Figure 1: Logic Model for Test Bed Evaluation

275x190mm (300 x 300 DPI)

Appendix 1

Participant payment

Participants taking part in the qualitative elements of the study will receive a voucher of £20 as a thank you in recognition of the time they have given to taking part in the evaluation. In the quantitative elements of the evaluation we are unable to offer any incentive because of the large numbers of participants.

Data storage

Personal data is being stored on a password safe university computer accessible only to the researchers. The research data is stored in an encrypted file using the software TrueCrypt.

All data is being stored securely in line with the Data Protection Act [15] and will be kept no longer than necessary.[16] Written data will be kept for a maximum of 10 years in a secure cabinet. Any electronic copies of data will be kept on the University server, which is a password protected system and will be encrypted. If electronic data requires transfer, this is done on an encrypted USB device. Any emails sent from participants (e.g. electronic diaries) are sent to a password secured email address that has been set up for the purpose of the study. Emails are saved electronically with any other electronic copies of data, with the email then being deleted. Where emails and interview/focus group data are printed for the purposes of analysis, they are stored with other written data in a secure cabinet. At the end of the study, the email account will be deleted. The paper copies of any participant contact details will be scanned and stored electronically, and paper copies destroyed.

Participant benefit

Participants have the direct benefit of using the technology during the two years of the Test Bed; however the Test Bed team recognise the need to make clear for those in Cohorts 1 and 4 that this is time-limited. Patients (particularly those in cohorts 2 and 3) for whom the intervention is a text messaging system or an app, will have access to these beyond the six month timeframe of the Test Bed programme. Given a key objective of the service is to enhance patient activation and ability to self-care, it is also anticipated that potential ongoing benefits will support older people with chronic health problems to remain in their own homes longer.

Patients and health professionals also have an opportunity to share directly with researchers their experiences of using and implementing this model of care. In this way, all participants can take an active part in shaping current and future healthcare services in place for older people with chronic conditions or dementia who are living at home.

Potential risk to participants

For patients participating in Phase 1 of the evaluation, there are no anticipated risks from participation in the project. All validated tools are validated for completion by proxy, and patients and family

For peer review only

members are therefore made aware that the patient can be supported to complete the surveys if required.

For patients taking part in Phase 2 of the evaluation research, no specific risks are anticipated. However, the following considerations have been made:

1. It is possible that a patient may become distressed by the presence of the researcher in their home during the observational interviews. To avoid this risk, the researcher will take care to check with the patient at the beginning of the interview to ensure they are happy to continue taking part in the study. The researcher will also do this at the end of the session and for the second interview.

2. If a patient should become distressed for any reason during an observational interview, the researcher will provide the patient with the contact details of different support networks for older people, as outlined in the participant information sheet. A member of the Test Bed hub will also be alerted should this be necessary, in order to assess whether a visit from a member of the hub is required.

Participants involved in the action learning meetings and the focus groups will be reminded that discussion in these settings is confidential and information shared (other than the shared and agreed action learning) should not be discussed with others outside of the meeting.

Withdrawal

Participants may choose to leave the study at any time and are assured that their data can be withdrawn up to four weeks after collection. The rationale for this four week time period is that, beyond four weeks, analysis will have begun. Those participating in focus groups are advised that, although every effort will be undertaken to remove their data should they request it during the timeframe, this may not always be possible. This is due to difficulties in identifying/attributing specific comments to individuals and because focus group data is often the outcome of group discussion, making it hard to distil to whom individual thoughts/comments should be attributed.

Researcher risk

There is limited risk to the quantitative researchers as they have very limited direct engagement with the participants (survey completion is largely through technology or telephone).

The qualitative researcher is interviewing participants in their own homes and is experienced in undertaking this type of fieldwork. The researcher is adhering to the University's lone worker policy. In order to minimise any potential risks, for each home visit conducted, the researcher identifies a 'buddy' (a member of staff within the University) with whom to remain in contact with: before the interview commences; when the interview has been terminated; and when the researcher has left the patient's home. The buddy is informed of the time and place of the interview, along with a contact telephone number.

Researchers within the team have the opportunity to debrief with other members of the team if required and have full access to counselling services if necessary.

References

For peer review only

15 Data Protection Act (1998) Data Protection Act. Available at: http://www.legislation.gov.uk/UKPGA/1998/29/contents [Accessed on 17 February 2017].

16 Bryman A. Social Research Methods, Sixth Edition. Oxford, UK: Oxford University Press 2016.

For peer review only

How effective and cost effective are innovative combinatorial technologies and practices for supporting older people with long-term conditions to remain well in the community? An evaluation protocol for an NHS Test Bed in Northwest England.

Journal:	*BMJ Open*
Manuscript ID	bmjopen-2017-017268.R3
Article Type:	Protocol
Date Submitted by the Author:	15-Jan-2018

Complete List of Authors:	Varey, Sandra; Lancaster University, Division of Health Research<br>Hernândez, Alejandra; Lancaster University<br>Palmer, Tom; Lancaster University, Department of Mathematics and Statistics ; University of Warwick, Warwick Medical School, Division of Health Sciences<br>Mateus, Ceu; University of Lancaster, Division of Health Research<br>Wilkinson, Joann; Lancaster University<br>Dixon, Mandy; Lancaster University<br>Milligan, Christine; Lancaster University

**Primary Subject Heading**:	Health services research
Secondary Subject Heading:	Geriatric medicine
Keywords:	Health technologies, older people, long-term conditions, patient activation, self-care

Title: How effective and cost effective are innovative combinatorial technologies and practices for supporting older people with long-term conditions to remain well in the community? An evaluation protocol for an NHS Test Bed in Northwest England.

Sandra Varey[1], Alejandra Hernández[1], Tom Palmer[2], Céu Mateus[3], Joann Wilkinson[1], Mandy Dixon[4] and Christine Milligan[1]

1. Centre for Ageing Research, Division of Health Research, Lancaster University, Lancaster LA1 4YG, United Kingdom

2. Department of Mathematics and Statistics, Lancaster University, Lancaster LA1 4YG, United Kingdom

3. Health Economics, Division of Health Research, Lancaster University, Lancaster LA1 4YG, United Kingdom

4. Lancaster Health Hub, Lancaster University, Lancaster LA1 4YG, United Kingdom

Correspondence to:

Professor Christine Milligan

Centre for Ageing Research

Division of Health Research

Lancaster University

Lancaster LA1 4YG

United Kingdom

Email: c.milligan@lancaster.ac.uk

Telephone: +44 1524 592128

Word count: 4591

For peer review only - http://bmjopen.bmj.com/site/about/guidelines.xhtml

Abstract

Introduction:
The Lancashire and Cumbria Innovation Alliance (LCIA) Test Bed is a partnership between National Health Services in England (NHS), industry (led by Philips) and Lancaster University. Through the implementation of a combination of innovative health technologies and practices, it aims to determine the most effective and cost effective ways of supporting frail older people with long-term conditions to remain well in the community. Among the Test Bed's objectives are to: improve patient activation and the ability of older people to self-care at home; reduce healthcare system utilisation; and deliver increased workforce productivity.

Methods and Analysis:
Patients aged 55 years and over are recruited to four cohorts defined by their risk of hospital admission, with long-term conditions including COPD, dementia, diabetes and heart failure. The programme is determined on an individual basis, with a range of technologies available. The evaluation is adopting a two-phase approach: Phase 1 includes a bespoke patient survey and a mass matched control analysis; and Phase 2 is using observational interviews with patients, and weekly diaries, action learning meetings and focus groups with members of staff and other key stakeholders. Phase 1 data analysis consists of a statistical evaluation of the effectiveness of the programme. A health economic analysis of its costs and associated cost changes will be undertaken. Phase 2 data will be analysed thematically with the aid of Atlas.ti qualitative software. The evaluation is located within a logic model framework, to consider the processes, management and participation that may have implications for the Test Bed's success.

Ethics and Dissemination:
The LCIA Test Bed evaluation has received ethical approval from the Health Research Authority and Lancaster University's Faculty of Health and Medicine Research Ethics Committee. A range of dissemination methods are adopted including deliberative panels to validate findings and develop outcomes for policy and practice.

Word count: 300

For peer review only

For peer review only - http://bmjopen.bmj.com/site/about/guidelines.xhtml

Strengths and limitations of this study

- The evaluation is considering both the effectiveness, in terms of measures such as hospital admissions, and cost effectiveness of the Test Bed
- The protocol evaluates the potential for patient activation through health technology
- Observational interviews with patients in their own homes enable insights into how older people experience and engage with the technology
- For both the Test Bed participants and controls the evaluation has to use proxy measures of the costs incurred to the NHS
- Due to the timescale of the overall programme, it is not possible to assess the longer-term impact of the programme

Key words

Health technologies; older people; long-term conditions; patient activation; self-care.

For peer review only

For peer review only - http://bmjopen.bmj.com/site/about/guidelines.xhtml

INTRODUCTION

In the UK, increasing numbers of older people are living with long-term conditions including diabetes, chronic obstructive pulmonary disease (COPD), heart failure and dementia.[1] This is contributing to increased pressure on health and social care systems including accident and emergency services, which in turn increases demand on the number of hospital beds.[2] At the same time, local authorities are experiencing a reduction in the funding available to provide social care.[3]

Concerns about this so-called care-gap and our future ability to cope with the growing numbers of older people with long-term conditions underpins governmental drivers to develop new models of care within Vanguard sites[1] that are cost-effective and which facilitate older people's ability to better self-manage their care needs at home. The NHS England Test Bed programme has thus been designed to encourage the trialling of new models of care that are supported by health technologies across a number of areas in the UK.

This paper reports on the protocol for the evaluation of one such programme, the Lancashire and Cumbria Innovation Alliance (LCIA) Test Bed in the north-west of England. The programme commenced on 1st April 2016 and is due to be completed on 30th June 2018.

The LCIA Test Bed initiative

The LCIA Test Bed[2] is a partnership between the NHS, industry (led by Philips) and Lancaster University, and is one of seven Test Beds located across England. Two neighbouring Vanguard sites, part of the Lancaster Health Hub (an established NHS/University partnership comprising ten local organisations), will deliver the Test Bed. The Test Bed's aims and objectives are presented below.

Test Bed Aims:
To determine the most effective and cost effective ways of supporting frail older people with dementia and other long-term conditions (LTCs) to remain well in the community and avoid unnecessary hospital admissions.

Test Bed Objectives:
To use a combinatorial range of technologies and services to:

- better support frail older people, older people living with long-term conditions;
- improve patient activation[3] and the ability of older people with a range of LTCs to self-care at home;
- improve health awareness and outcomes for older people with LTCs;
- reduce healthcare system utilisation and increase productivity within the healthcare workforce.

---

[1] In 2015, the National Health Service in England selected 50 vanguard sites to take a lead on the development of new integrated care models designed to act as blueprints for the future of the NHS. Complete redesign of whole health and care systems are being considered. The hope is that the vanguards will provide inspiration to the rest of the health and care system across the UK (NHS England, 2017).[4] For more information about NHS Vanguards, visit: https://www.england.nhs.uk/ourwork/new-care-models/vanguards/

[2] For more information about the LCIA Test Bed, visit: https://www.england.nhs.uk/ourwork/innovation/test-beds/innovation-alliance/

[3] For more information about the NHS definition of patient activation, visit: https://www.england.nhs.uk/ourwork/patient-participation/self-care/patient-activation/pa-faqs/

For peer review only - http://bmjopen.bmj.com/site/about/guidelines.xhtml

Over two years, the Test Bed is implementing a combination of innovative technologies and practices aimed at supporting these aims and objectives. The clinical challenge is to reduce hospital admissions, create capacity, reduce overall costs, minimize other health and social care usage, and maximize patient outcomes for a targeted population of older people (defined in this Test Bed as those aged 55 years and over) who suffer a range of long term conditions including dementia. These LTCs present a major challenge for the area given its dispersed population, and innovative solutions are urgently required.

The main technology partner is Philips who, together with a number of small and medium sized enterprises (SMEs), social enterprises, and voluntary organisations, is working with the Test Bed to introduce a health technology-enabled supported self-care programme.[4]

The two Vanguard sites include the Fylde Coast and North Lancashire, with the technologies being provided in addition to the new models of care being implemented by these two sites. The Fylde Coast Vanguard[5] is drawing on the Extensive Care model, which brings together a range of services in the same location to achieve a co-ordinated team of healthcare professionals, with patients each allocated a wellbeing support worker.[5] This model focuses on multispecialty community providers, moving specialist care out of hospitals and into the community.[6] The North Lancashire Vanguard, Better Care Together, sees healthcare professionals working together in partnership to help people manage their own health conditions.[7] This model focuses on integrated primary and acute care settings, joining up GP, hospital, community and mental health services.[8] Further details of these new models of care are set out in the NHS England 'Five Year Forward View' [9] and by the Nuffield Trust [10].

As part of the LCIA Test Bed programme, these existing services are being combined with innovative technologies and support for older people with LTCs including COPD, Heart Failure, Diabetes and Dementia. For those with lower levels of risk in cohort 3 (see Table 1), asthma and hypertension will also be included. The focus of this Test Bed is to empower patients to actively manage their health conditions and change behaviour through the provision of appropriate technologies, coaching and education.

A core part of the programme also focuses on increasing involvement of the third sector and community organisations in providing support for the Test Bed model, and understanding the impact of the service on staff and key stakeholders.

Core outcomes for the evaluation

The Test Bed focuses on a combinational approach to technologies. Hence, rather than focusing on the impact of any single technology, the Test Bed evaluation is focusing on how health technologies as an addition to existing Vanguard services can improve and promote self-care at home and reduce hospital admissions for older people with LTCs.

The evaluation is focusing on three primary outcomes:

1. The extent to which supported self-care telehealth technology can improve patient outcomes, through reduced hospital admissions, medication, GP visits, home visits and community

[4] Cambridge Cognition Limited; Good Things Foundation; Intelesant Limited; MKS Solutions Limited; Simple Shared Healthcare Limited; and uMotif Limited.

[5] The Fylde Coast Vanguard encompasses NHS Blackpool CCG, NHS Fylde & Wyre CCG and Blackpool Teaching Hospitals.

For peer review only - http://bmjopen.bmj.com/site/about/guidelines.xhtml

service use;

2. The extent to which supported self-care telehealth technology can improve patient activation for frail older people with LTCs;

3. The cost effectiveness of the programme and how it might be scaled up to provide better value for patients and taxpayers.

Secondary outcomes include:

1. Identification of the strengths and weaknesses of the programme and how these may be built on or addressed;

2. Assessment of patient/staff perceptions of how the new service improves on existing services and makes best use of voluntary and community services;

3. Assessment of the impact of the new service within the healthcare workforce in terms of communication between care teams, productivity, capacity, co-ordination of care, and work satisfaction.

To achieve this, we are:

a) Using quantitative and qualitative measures to examine benefits to patients and patient outcomes as identified above;

b) Undertaking an economic evaluation of the impact of adding a combination of technologies to support and promote self-care in the provision of healthcare in comparison to standard care with Cohorts 1 and 2 (see Table 2 below);

c) Drawing on a Logic Model to demonstrate how to construct the service as a scalable model that might provide better value to patients and taxpayers.

METHODS AND ANALYSIS

Ethical approvals

The overall evaluation involves two distinct phases: Phase 1 involves collection of survey data designed to evaluate any change in health and health-related behaviours in patients using the combinatorial technologies; and Phase 2 involves understanding the impact and experiences of patients using the technologies, and the impact on working practices amongst members of staff involved in the delivery of the technologies. Following discussion with the Health Research Authority (HRA) and the Research and Development Directors of the NHS Trusts involved in the programme, it was agreed that Phase 1 was Service Evaluation and as such did not require HRA approvals. Phase 2 however was deemed by the HRA to be gathering new data and was thus defined by them as ‘research’, therefore requiring HRA approvals (IRAS Project ID: 208395). We received ethics approval from Lancaster University’s Faculty of Health and Medicine Research Ethics Committee for Phase 1 (Reference: FHMREC16025). Further details about the two phases are provided below.

For peer review only - http://bmjopen.bmj.com/site/about/guidelines.xhtml

Phase 2 of the evaluation has also been adopted onto the National Institute for Health Research portfolio.[6]

Sampling and recruitment

Patients are being recruited to four cohorts defined by their risk of hospital admission. The long-term conditions are noted above and are outlined in Table 1.

Table 1: Recruitment criteria for the four LCIA Test Bed cohorts

Cohort	Age	Risk of hospital admission	Long-term conditions
Cohort 1	Aged 55 years or over	>25%	COPD, HF
Cohort 2		>10% and <25%	
Cohort 3		<10%	Diabetes, Asthma, CHD, Hypertension
Cohort 4		N/A	Early stage dementia (ACE-III assessment tool)

Participants for cohorts 1, 2 and 3 are being recruited to the Test Bed through the Vanguard services to which they are already assigned. As illustrated in Table 1, Cohort 4 is focusing specifically on patients with early stage dementia. Potential participants for Cohort 4 are being recruited through the Memory Assessment Service (MAS) in the Fylde Coast and North Lancashire. In the diagnosis of dementia, these services use the Addenbrooke's Cognitive Examination-III (ACE-III) assessment tool to focus on five cognitive domains. People who have received a recent diagnosis of mild dementia by a consultant psychiatrist following their comprehensive assessment with the MAS are deemed as having fulfilled the entry criteria of mild dementia. For these patients, the ACE-III or Mini-ACE has been completed as part of their cognitive assessment at the MAS clinic, the results of which have aided the formulation of the dementia diagnosis. Only patients judged by a clinician to have mild dementia are invited to take part in the Test Bed.

All patients consenting to the take part in the Test Bed and receiving the technology are required to participate in some aspect of Phase 1 of the service evaluation and will sign a consent form. Specifically, primary outcomes 1, 2, and 3 will be evaluated for cohorts 1 and 2; and primary outcome 1 and 2 will be evaluated for cohorts 3 and 4. Participation in Phase 2, the qualitative phase of the evaluation is optional. Patients indicating an interest in participating in Phase 2 are being consented separately through a two-stage consent process.

The sample size for recruitment to each cohort has been informed by population and risk data for the area, along with the methodology used in a previous study.[11,12] As Table 2 illustrates, the overall aim of the Test Bed is to recruit 1600 patients; however, given the anticipated challenges of recruitment and potential attrition due to the nature of the patient group, we estimate an overall recruitment and retention figure of 60% (overall N=960).

Table 2: LCIA Test Bed cohort sample sizes (*combined over a population of app. 310,000 people)[7]

[6] NIHR Portfolio reference number is the same as the IRAS number (IRAS Project ID 208395)
[7] See Acknowledgements

For peer review only - http://bmjopen.bmj.com/site/about/guidelines.xhtml

Cohort	Percent of the total population*	Total patients	Percent of frail and elderly patients	Percent of patients with dementia	Number of patients estimated to be appropriate for the service	Percent of appropriate patients within cohort that will be recruited within the Test Bed period	Minimum number of patients recruited within Test Bed period	Estimated number of patients by the end of the Test Bed period
**1** (age>55, risk>25% & 1 LTC)	1.70%	5,262	20%	3.5%	1,737	30%	521	500
**2** (age>55, 10%>risk<25 % & 1 LTC)	3.00%	9,286	4%	1.1%	3,064	15%	460	500
**3** (age>55, 10%<risk 1 of a broader set of LTCs)	9.20%	28,477	2%	1.6%	9,398	5%	470	500
**4** (diagnosis of dementia)	0.79%	2,430		100.0%	810	10%	81	100

Allowing for a phased increase in recruitment, the overall recruitment is estimated at approximately 60-70% of the figures identified in Table 2. This will provide sufficient data from cohorts 1 and 2 to demonstrate a change in hospital admissions (a primary outcome). Cohorts 3 and 4 will not be assessed for this outcome, although other measures of wellbeing, patient activation, and loneliness will be used to describe these cohorts. The secondary outcomes are being supported by observational interviews across all four cohorts.

The programme

The patient interface varies across the four cohorts, and the technologies available to people within the different cohorts are presented in Table 3. A patient's programme is determined on an individual basis and is informed by a number of factors, including health condition(s), personal preferences and other needs. Some patients are using different technologies at different periods of time within the six month programme.

Table 3: Technologies available to each cohort

Cohort	Company	Technology	Description
1 & 4	Philips	Motiva[8]	A telehealth platform which operates through a tablet or TV set top box interface that connects wirelessly to a range of tele-monitoring equipment in the home (e.g. wireless weighing scales, blood pressure meters, pulse oximeters and thermometers).
	MKS Solutions Limited	Speakset[9]	A video calling system that connects a health professional with a patient through the patient's TV in their own home.

[8] http://www.philips.co.uk/healthcare/solutions/enterprise-telehealth/home-telehealth
[9] http://www.speakset.com

For peer review only - http://bmjopen.bmj.com/site/about/guidelines.xhtml

	NHS Stoke-on-Trent Clinical Commissioning Group's Licence	Florence/NHS Simple[10]	A short message communication software providing a reminder/communication text messaging service for patients.
1, 2 & 3	Cambridge Cognition Limited	CANTAB Mobile[11]	An assessment tool designed for healthcare professionals to identify the earliest signs of clinically significant memory impairment. The assessment comprises three tests: the Paired Associates Learning (PAL) test to assess episodic memory; the Geriatric Depression Scale (GDS) to identify signs of depression and an Activities of Daily Living questionnaire (ADL) to assess functionality in daily life.
2	Intelesant Limited	Intelesant[12]	A mobile app to support daily management of COPD through SMS text messaging alerts for coaching, prompts, and reminders. It incorporates the individual's action plan in response to changes in symptoms.
	Philips	Personal Blood Pressure Cuff[13]	A blood pressure monitor that works in conjunction with the Philips Suite App to enable a patient to record their blood pressure.
2 & 4	Philips	Health Watch[14]	A health watch that tracks heart rate and other cardio condition metrics, monitors activity, sleeping patterns, and nutrition behaviour (not a sports watch). It is best paired with a smartphone running the Philips Suite App.
3	uMotif Limited	uMotif[15]	A health app available as a mobile app, digital tool or wearable device to capture data through a graphical interface and help patients track and understand their health and symptoms. It provides a health report, connects to other wearable devices, sends medication reminders, and keeps track of regular tasks and daily activities.
4	National Museums Liverpool	House of Memories[16]	A health app that enables people living with dementia and their carers/families to keep a record of objects and experiences from the past. Patients and/or carers can create their own memory tree, memory box or memory timeline.

In summary, the LCIA Test Bed provides a structured programme of case management, monitoring, education and coaching supported by a clinical hub. It thus involves clinical and non-clinical teams, community involvement and community feedback. Good Things Foundation[17] is providing support to patients regarding their digital skills to help them use the innovator technologies.

[10] http://www.simple.uk.net
[11] http://www.cambridgecognition.com
[12] http://www.intelesant.com
[13] http://www.philips.co.uk/c-m-hs/health-programs/upper-arm-blood-pressure-monitor
[14] http://www.philips.co.uk/c-m-hs/health-programs/health-watch
[15] http://www.umotif.com
[16] http://www.liverpoolmuseums.org.uk/learning/projects/house-of-memories/
[17] http://www.goodthingsfoundation.org

For peer review only - http://bmjopen.bmj.com/site/about/guidelines.xhtml

Evaluation design

A requirement of this evaluation[18] was that it adopt a rapid cycle approach, to enable the delivery of the programme to be adapted and amended in the light of feedback received from patients and clinicians. As a result, a randomised controlled trial design was deemed unsuitable.

To address the core aims and objectives outlined above and the NHS England requirements, the evaluation was designed as a longitudinal, mixed method evaluation, to include:

Phase 1:

1. A bespoke patient survey incorporating validated tools;
2. A mass matched control analysis.

Phase 2:

3. Phased observational interviews with patients;
4. Weekly diaries, action learning meetings and focus groups with staff and other key stakeholders.

The two phases of the evaluation design are adopting different sampling and recruitment strategies, along with methods, as set out below. Each has its own participant information sheets, consent forms and, where applicable, schedules (e.g. for the interviews and focus groups). A separate flyer has been designed for patients with dementia, providing information about the study in an informative and accessible manner, drawing on images and large font. In all instances, it will be made clear to patients that they can be supported by a carer or other family member should they wish. It is anticipated that all participants in cohort 4 (mild dementia) will be accompanied by a carer or other family member.

All materials developed for the evaluation and submitted to ethical review were shared and informed through discussion with: patient by experience groups (including those with dementia); a clinical reference group; and an evaluation advisory board. Feedback and comment from these three groups was taken into account in finalising the documents submitted for ethics approval.

1. The patient survey

Recruitment and informed consent

In line with referral to the service, recruitment is being staggered over the programme period. Only patients judged by a clinician to have an acceptable level of cognitive function to give informed consent are invited to take part in the Test Bed. The impact of the programme on each participant is being evaluated over a six month period. Final recruitment to the evaluation will thus be at the end of month 15, to allow for the six month programme and a three-month period for final analyses. All patients recruited into the Test Bed and receiving the service are included in the quantitative (service evaluation) stage, and are completing the Phase 1 consent form. Patients in Phase 1 are completing a baseline, mid (12 weeks) and endpoint (24 weeks) survey during the six month programme.

Methods

The data analysis consists of a statistical evaluation of the effectiveness of the programme and a health economic analysis of its costs and associated cost changes for Cohorts 1 and 2. The evaluation is

[18] Stipulated by NHS England

For peer review only - http://bmjopen.bmj.com/site/about/guidelines.xhtml

collecting anonymised information for each participant concerning: age, gender, ethnicity, living arrangements, education completed, and use of health care services in the four weeks before completing the questionnaire such as hospital services (inpatient care, outpatient visits, and A&E visits), primary care services, social care services, and medicines. In all cohorts, validated instruments for use with older people to assess health related quality of life, health and wellbeing and patient activation are being used (each of can be completed by proxy if required): Warwick-Edinburgh Mental Wellbeing Scale (WEMWBS), EQ-5D-5L, Patient Activation Measure (PAM)13, and the De Jong Gierveld Loneliness Scale. These tools are reapplied at weeks 12 and 24.

The evaluation team are working with the technology innovators[19] to minimise the demands of the evaluation on patients. Participants are also being offered the option of completing the questionnaires on paper or by telephone.

Drawing on HES data, the primary outcomes for cohorts 1 and 2 will be evaluated against a matched control group (3 controls to 1 Test Bed patient). This will be coordinated in conjunction with the Midlands and Lancashire Commissioning Support Unit.

For Cohorts 1 and 2, the cost-effectiveness analysis of the programme will assess the costs per hospital admission avoided. For the Test Bed patients and for the control group the mean of hospital admissions will be estimated. The primary outcome is the difference between the change in the two groups which will be estimated using linear regression adjusted for the matching covariates. Ninety-five percent confidence intervals will be reported for all estimated coefficients. A sensitivity analysis for key parameters will be undertaken.

2. The mass control group

The evaluation team will match 3 control subjects per programme participant for Cohorts 1 and 2, the controls being sampled from the same Vanguard Sites as those on the Test Bed. In terms of contamination between control and Test Bed groups, obtaining control data from the Commissioning Support Unit ensures no controls are also members of the Test Bed. The matching will be performed using the following variables: predicted probability (risk) of hospital admission, long-term condition, sex, and age. The predicted probability of hospital admission for each participant and control will be calculated using the Combined Predictive Model.[13] The matching will be performed using a standard multivariate matching algorithm (Mahalanobis distance matching). The aim of matching on these variables is to obtain a control group which has similar characteristics for these variables compared to the programme participants. This should help minimise any possible confounding effects of these variables.

The statistical analysis is conducted using the statistical software Stata (version 14).

3. Phased observational interviews with patients

Recruitment and informed consent

Patient participation in Phase 2 of the evaluation is optional and participants are asked to indicate their preference for this in the Phase 1 consent form. Based on past experience, it is anticipated that more patients will indicate a willingness to take part in the second phase than will be required. Where

[19] The term 'innovators' has been coined by NHS England for the purposes of the Test Beds and refers to technology companies deemed to be working at the cutting edge of digital health design and development.

For peer review only - http://bmjopen.bmj.com/site/about/guidelines.xhtml

possible, we are seeking to purposively sample on the basis of: chronic health condition(s) (cohorts 1-3); age (55-64; 65-74; 75-84; ≥85); gender; ethnicity; postal address (based on the ONS deprivation scale); and co- versus lone dwelling. Ten to fifteen patients are being recruited from each cohort to participate in the in-depth qualitative phase of the evaluation (N = 40-60).

Those participating in Phase 2 are being consented at the time of their first interview. A separate flyer has been designed for patients with dementia, to provide information about the Test Bed in an informative and accessible manner. To ensure informed consent and that each participant understands the consent form, the researcher discusses each aspect of the consent form with each participant before it is signed.

Ethical approval was received from the HRA for an alternative consent form with a large font size, and for which clauses on the consent from can be cut into strips, with one clause per strip of paper. This enables the researcher to go through each clause with the participant, focusing on one strip of paper at a time. Once the participant understands the clause clearly, they are asked to initial the clause discussed. This option makes the information in the consent form more manageable for the patient and, prior to ethical approval, was discussed and approved by the Lancaster patient experience group (linked to the NHS). When consenting a person to Cohort 4, the researcher talks through the consent form with the patient and in the presence of a family member, friend or carer.

Methods

Two observational interviews are being undertaken with each participant in their own home over the six month period of the programme: a) in the first month of participating in the programme; and b) during the final month 6. The home-base setting is important as it facilitates a better understanding of participants' health status, health knowledge and activation prior to their participation in the programme, how they engage with the service and use the technology within their own homes at the outset and whether this changes over time. This approach is specifically designed to gain a better understanding of: how participants engage with and experience the technology and Fylde Coast Vanguard / Better Care Together services; how this may influence patient activation and self-management of care; any barriers to improving self-care; whether the programme can increase their sense of empowerment and independence; service satisfaction; and overall quality of life.

4. Understanding the impact on staff and key stakeholders: diaries, action learning meetings and focus groups

In the early stage of the Test Bed, interviews and focus groups will be conducted with key stakeholders to determine the lessons learned in the process of operationalising the Test Bed. This data will begin to inform and help map out the logic model (see below). Informed consent will be taken for all participants.

All key stakeholders and service providers involved in the delivery of the service are also invited to complete brief weekly diaries and participate in regular 'action learning' meetings, informed by the diary data, as part of a rapid cycle review process. Here, members of the evaluation team meet regularly with key stakeholders involved in the implementation and delivery of the service to reflect on shared learning, agreed action and the impacts of change. This includes technology innovators, members of staff from the Test Bed hubs, voluntary sector providers and others. Numbers for these meetings are anticipated to be around 20 people, but should numbers increase beyond this we will ask stakeholders to nominate representatives to participate in the meetings and act as two-way conduits for the learning from their particular stakeholder group.

For peer review only - http://bmjopen.bmj.com/site/about/guidelines.xhtml

Action learning meetings will take place at three-monthly intervals and will involve a cycle of discussion, shared learning and reflection and agreed action to be taken forward regarding the operation, delivery and effectiveness of the service. Agreed action from these meetings will build on this cycle of learning and assessment of the service throughout the Test Bed period. To facilitate discussion at these meetings, key stakeholders are asked to complete weekly diaries documenting their experiences of the service, including what works well and less well, and any observations of patients' responses to the service (time spent/support need/technical difficulties). Diaries are brief and simple to complete and are designed to feed into the action research meetings and to fine-tune themes for the focus groups and deliberative panels. The diaries are submitted electronically with participants being encouraged to keep a personal copy as an 'aide memoire' prior to each action learning meeting.

Toward the end of the evaluation we will also hold four focus groups (N=6-10 participants per focus group) with key stakeholders involved in the delivery of the service. Our objective here is to not only recruit those directly involved in delivering the service to older participants but also those organising and managing it. Recruitment to the focus groups will be split evenly across the two sites. The aim here will be to gain an in-depth understanding of how Fylde Coast Vanguard/Better Care Together services with the technology is impacting on working practices. The focus groups will also explore the extent to which the programme may help to increase: communication between the care teams; productivity; capacity and co-ordination of care; and overall work satisfaction.

For ease of reference, a summary of the overall evaluation methods is set out in Table 4 below.

Table 4: Summary of evaluation methods being applied to Test Bed participants

Participants	Survey	Observational interview	Focus groups	Diaries	Action Learning meetings	Deliberative panels
Patients (with carers as appropriate)	Yes (3 over 6 months)	Yes (2 over 6 months)	N/A	N/A	N/A	Sample
Staff delivering service	N/A	N/A	Yes	Yes	Yes	Sample
Other key stakeholders (technology innovators, voluntary sector, etc.)	N/A	N/A	Yes	N/A	Yes	Sample
Managers/administrators	N/A	N/A	Yes	N/A	N/A	Sample

ETHICS AND DISSEMINATION

Audio recording

All qualitative data is being audio recorded using an encrypting digital voice recorder. In the process of transcription, names of participants are anonymized and any identifying features removed or coded. These points are included in the consent forms and are discussed with each participant before the interview/action research meeting/focus group/deliberative panel. All audio files will be deleted from the recorder once data analysis is complete.

Data analysis

The qualitative data will be analysed thematically with the aid of Atlas.ti qualitative software. Initial identification of emerging themes and initial coding will be undertaken by the field researcher then shared and agreed during evaluation team data analysis workshops. This will allow for the verification of the coding framework and enable the evaluation team to develop an analysis of the situation and

For peer review only - http://bmjopen.bmj.com/site/about/guidelines.xhtml

understand the phenomenon being explored.[14] The major codes emerging from the data will be presented to the deliberative panels for fine-tuning and final verification.

Appendix 1 contains information regarding participant payment, data storage, participant benefit, potential risk to participants, withdrawal and researcher risk.

Deliberative panels

In the final three months of the evaluation, we will hold two deliberative panels (one per Vanguard site: N=15-20 per panel) with key stakeholders to include: older participants; members of the Vanguard care teams; key stakeholders from clinical and community settings, and social care; innovators; and senior managers and commissioners from the Test Bed sites.

Deliberative panels are designed to validate and fine-tune outcomes by producing an informed and collective view resulting from deliberation. They offer a bottom-up approach that can shift evaluation findings into outcomes related to policy, practice and guidance for employers. To achieve this, draft findings emerging from the data analyses will be presented with the aim of drawing on the various expertise of the participants.

In this case, it will help to identify and validate those key components of the programme that will comprise a new model of care that may be scaled up for wider roll-out and will help to finalise the evaluation recommendations. The deliberative panels will also be used to begin preliminary discussions of how this model might form the basis of a joint health and social care strategy for telehealth/telecare. Finally, the panels will help us to fine-tune the Logic Model through a process of 'backward mapping' (see below).

The Logic Model framework

The evaluation is located within a Logic Model framework, enabling us to understand the impact of the programme. It also enables us to consider the processes, management and participation that may have implications for the Test Bed's success. In developing the Logic Model, we are considering the following key elements:

- Activities – the focus of our model is the New Models of Care with Technology programme. The programme of work is considered in its context, including the partners of the project and the policies and procedures that are in place.
- Inputs – including the financial, human, organisational, and material resources of the project. This includes the following stakeholders: older adults taking part in the project; Fylde Coast Vanguard and Better Care Together teams; clinicians; GPs; community nurses; other health professionals; and innovator, voluntary and community sector input.
- Outcomes – the desired and actual results of the project and a Logic Model that will support roll out and scalability.

The Logic Model has been designed as a processual model that will be populated using data gathered in the diaries and action research meetings. The elements identified above will be fine-tuned iteratively over the two years of the programme and finalised through a process of 'backward mapping' during the final deliberative panels. In this way, the final Logic Model, designed to assist with wider roll-out of the programme, will be developed by drawing on informed and collective views

For peer review only - http://bmjopen.bmj.com/site/about/guidelines.xhtml

resulting from ongoing iteration and deliberation involving all key stakeholders. Figure 1 provides an overview of the Logic Model being used for the Test Bed evaluation.

Dissemination

The findings of the evaluation will be disseminated in the following ways:

a) A final evaluation report which also contributes to the national evaluation;
b) Presentations to all key stakeholders within the Vanguard sites and NHS England;
c) Conference papers and posters presented at regional, national, and international conferences;
d) Publication in a range of high quality, international, peer-reviewed scientific journals;
e) Publication in a range of journals/publications relevant to service providers;
f) Dissemination of evaluation highlights through social media.

FULL REFERENCES

1 George J & Martin F (2016) Briefing paper: Living with long-term conditions. British Medical Association. Available at: https://www.bma.org.uk/-/media/files/pdfs/.../living-with-long-term-conditions.pdf [Accessed on 20 November 2017].

2 King's Fund, The (2016) Quarterly Monitoring Report, No. 21. Available at: http://qmr.kingsfund.org.uk/2016/21?_ga=1.123564811.1874139399.1487344226/ (November 2016) [Accessed on 20 November 2017].

3 Local Government Association, The (2016) 'Adult Social Care Funding: 2016 State of the Nation Report'. November 2016. Available at: http://www.local.gov.uk/documents/10180/7632544/1+24+ASCF+state+of+the+nation+2016_WEB.pdf/e5943f2d-4dbd-41a8-b73e-da0c7209ec12 [Accessed on 20 November 2017].

4 NHS England (2017) 'About vanguards'. Available at: https://www.england.nhs.uk/ourwork/new-care-models/vanguards/about-vanguards/ [Accessed on 20 November 2017].

5 NHS Blackpool Clinical Commissioning Group (2017) 'Extensive Care'. Available at: http://blackpoolccg.nhs.uk/local-services/new-models-of-care/extensive-care/ [Accessed on 20 November 2017].

6 NHS England (2017) 'Multispecialty Community Provider Vanguards'. Available at: https://www.england.nhs.uk/ourwork/new-care-models/vanguards/care-models/community-sites/ [Accessed on 20 November 2017].

7 Bay Health and Care Partners (2017) 'Guide to Better Care Together'. Available at: http://clinicalimages.web.furness.net/files/BCT-Guide-13Jul.pdf [Accessed on 20 November 2017].

8 NHS England (2017) 'Integrated Primary and Acute Care Systems Vanguards'. Available at: https://www.england.nhs.uk/ourwork/new-care-models/vanguards/care-models/primary-acute-sites/ [Accessed on 20 November 2017].

9 NHS England (2017) 'Five Year Forward View'. Available at:

For peer review only - http://bmjopen.bmj.com/site/about/guidelines.xhtml

For peer review only

https://www.england.nhs.uk/wp-content/uploads/2014/10/5yfv-web.pdf [Accessed on 20 November 2017].

10 Nuffield Trust (2017) 'New Models of Health Care Delivery'. Available at: https://www.nuffieldtrust.org.uk/our-priorities/new-models-of-health-care-delivery [Accessed on 20 November 2017].

11 van Berkel C, Smith M, Horsfield D. et al. Evidence for supported self-care at scale: A population approach to evaluating technology enabled support for long term condition management. Liverpool, UK: Philips and Liverpool Clinical Commissioning Group 2016.

12 van Berkel C, Smith M, Horsfield D. et al. Evidence for supported self-care at scale. *International Journal of Integrated Care* 2016 16(5) DOI: dx.doi.org/10.5334/ijic..2595

13 Wennberg D, Siegel M, Darin B, et al. Combined Predictive Model: Final report and technical documentation. London, UK: The King's Fund 2006.

14 Bowling A. Research Methods in Health: Investigating Health and Health Services, Fourth Edition. Maidenhead, UK: Open University Press 2014.

AUTHORS' CONTRIBUTIONS

Sandra Varey is the principal author and wrote the majority of the manuscript. Alejandra Hernández, Tom Palmer, Céu Mateus, Joann Wilkinson, Mandy Dixon and Christine Milligan are co-authors and each contributed to the editing and revision of the manuscript through several iterations. Christine Milligan is the Principal Investigator for the evaluation and is the corresponding author.

FUNDING STATEMENT

This work is supported by NHS England.

COMPETING INTERESTS STATEMENT

The authors declared no potential conflicts of interest with respect to the research, authorship, and/or publication of this article.

Acknowledgements

The authors wish to thank Amanda Thornton, the Patient by Experience groups, the Clinical Reference group and the LCIA Test Bed Evaluation Advisory Board for their helpful feedback on the initial design of the protocol materials.

In the original bid documentation, Philips contributed to the development of Table 2 presented in this paper.

For peer review only - http://bmjopen.bmj.com/site/about/guidelines.xhtml

Figure legends

Figure 1: Logic Model for Test Bed Evaluation

For peer review only

For peer review only - http://bmjopen.bmj.com/site/about/guidelines.xhtml

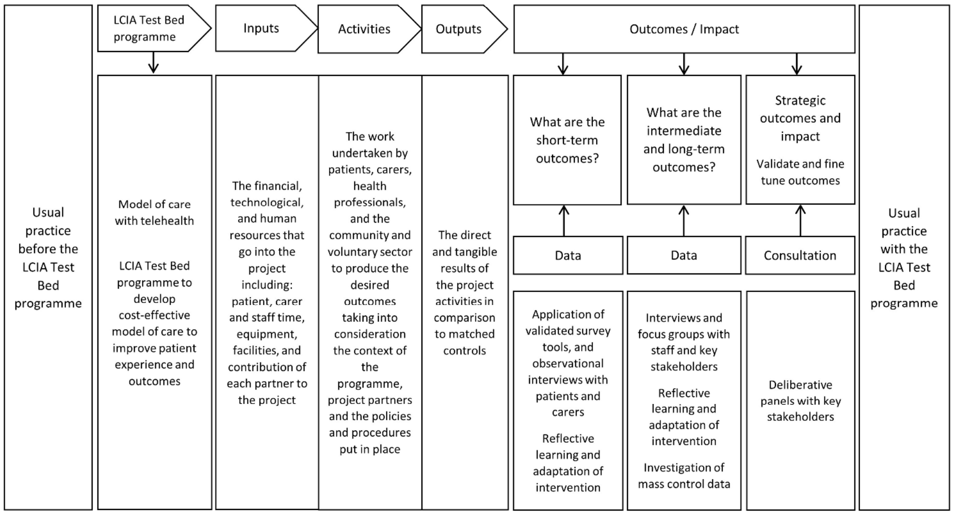

Figure 1: Logic Model for Test Bed Evaluation

275x190mm (300 x 300 DPI)

Appendix 1

Participant payment

Participants taking part in the qualitative elements of the study will receive a voucher of £20 as a thank you in recognition of the time they have given to taking part in the evaluation. In the quantitative elements of the evaluation we are unable to offer any incentive because of the large numbers of participants.

Data storage

Personal data is being stored on a password safe university computer accessible only to the researchers. The research data is stored in an encrypted file using the software TrueCrypt.

All data is being stored securely in line with the Data Protection Act [15] and will be kept no longer than necessary.[16] Written data will be kept for a maximum of 10 years in a secure cabinet. Any electronic copies of data will be kept on the University server, which is a password protected system and will be encrypted. If electronic data requires transfer, this is done on an encrypted USB device. Any emails sent from participants (e.g. electronic diaries) are sent to a password secured email address that has been set up for the purpose of the study. Emails are saved electronically with any other electronic copies of data, with the email then being deleted. Where emails and interview/focus group data are printed for the purposes of analysis, they are stored with other written data in a secure cabinet. At the end of the study, the email account will be deleted. The paper copies of any participant contact details will be scanned and stored electronically, and paper copies destroyed.

Participant benefit

Participants have the direct benefit of using the technology during the two years of the Test Bed; however the Test Bed team recognise the need to make clear for those in Cohorts 1 and 4 that this is time-limited. Patients (particularly those in cohorts 2 and 3) for whom the intervention is a text messaging system or an app, will have access to these beyond the six month timeframe of the Test Bed programme. Given a key objective of the service is to enhance patient activation and ability to self-care, it is also anticipated that potential ongoing benefits will support older people with chronic health problems to remain in their own homes longer.

Patients and health professionals also have an opportunity to share directly with researchers their experiences of using and implementing this model of care. In this way, all participants can take an active part in shaping current and future healthcare services in place for older people with chronic conditions or dementia who are living at home.

Potential risk to participants

For patients participating in Phase 1 of the evaluation, there are no anticipated risks from participation in the project. All validated tools are validated for completion by proxy, and patients and family

For peer review only

members are therefore made aware that the patient can be supported to complete the surveys if required.

For patients taking part in Phase 2 of the evaluation research, no specific risks are anticipated. However, the following considerations have been made:

1. It is possible that a patient may become distressed by the presence of the researcher in their home during the observational interviews. To avoid this risk, the researcher will take care to check with the patient at the beginning of the interview to ensure they are happy to continue taking part in the study. The researcher will also do this at the end of the session and for the second interview.

2. If a patient should become distressed for any reason during an observational interview, the researcher will provide the patient with the contact details of different support networks for older people, as outlined in the participant information sheet. A member of the Test Bed hub will also be alerted should this be necessary, in order to assess whether a visit from a member of the hub is required.

Participants involved in the action learning meetings and the focus groups will be reminded that discussion in these settings is confidential and information shared (other than the shared and agreed action learning) should not be discussed with others outside of the meeting.

Withdrawal

Participants may choose to leave the study at any time and are assured that their data can be withdrawn up to four weeks after collection. The rationale for this four week time period is that, beyond four weeks, analysis will have begun. Those participating in focus groups are advised that, although every effort will be undertaken to remove their data should they request it during the timeframe, this may not always be possible. This is due to difficulties in identifying/attributing specific comments to individuals and because focus group data is often the outcome of group discussion, making it hard to distil to whom individual thoughts/comments should be attributed.

Researcher risk

There is limited risk to the quantitative researchers as they have very limited direct engagement with the participants (survey completion is largely through technology or telephone).

The qualitative researcher is interviewing participants in their own homes and is experienced in undertaking this type of fieldwork. The researcher is adhering to the University's lone worker policy. In order to minimise any potential risks, for each home visit conducted, the researcher identifies a 'buddy' (a member of staff within the University) with whom to remain in contact with: before the interview commences; when the interview has been terminated; and when the researcher has left the patient's home. The buddy is informed of the time and place of the interview, along with a contact telephone number.

Researchers within the team have the opportunity to debrief with other members of the team if required and have full access to counselling services if necessary.

References

For peer review only

15 Data Protection Act (1998) Data Protection Act. Available at: http://www.legislation.gov.uk/UKPGA/1998/29/contents [Accessed on 17 February 2017].

16 Bryman A. Social Research Methods, Sixth Edition. Oxford, UK: Oxford University Press 2016.

For peer review only
